# Comparison of MAKO robotic-assisted and manual unicompartmental knee arthroplasty: a meta-analysis of radiographic precision and short-term functional results

**DOI:** 10.1007/s11701-026-03259-y

**Published:** 2026-03-02

**Authors:** Changjiao Sun, Xijiu Zhao, Qi Ma, Xiaofei Zhang, Jiawang Lou, Xu Cai

**Affiliations:** 1 Orthopaedics and Sports Medicine Center, Beijing Tsinghua Changgung Hospital, School of Clinical Medicine, Tsinghua Medicine, Tsinghua University, Beijing, P.R. China; 2 Institute of Orthopaedics and Sports Medicine, Tsinghua Medicine, Tsinghua University, Beijing, P.R. China; 3 Beijing Key Laboratory of Multimodal Intelligent Diagnosis and Therapy for Bone and Joint Injuries & Diseases, Beijing, P.R. China; 4 Department of Clinical Epidemiology and Biostatistics, Beijing Tsinghua Changgung Hospital, School of Clinical Medicine, Tsinghua Medicine, Tsinghua University, Beijing, P.R. China; 5 Beijing MEDERA Medical Group, Beijing, P.R. China

**Keywords:** Unicompartmental knee arthroplasty, Robotic-assisted, Meta-analysis, Mako

## Abstract

Background: This study sought to evaluate whether the MAKO robotic system offers superior radiographic and functional advantages over traditional techniques in unicompartmental knee replacement (UKA). Methods: A systematic literature search was performed through October 2025 across multiple electronic platforms, such as PubMed, Web of Science, Cochrane Library, Embase, Scopus, ClinicalTrials.gov, China National Knowledge Infrastructure (CNKI), Wanfang, China Biology Medicine Disc (CBM), and China Science and Technology Journal (CSTD). A total of 8,924 UKAs from 22 studies were included. Results: Findings indicated that MAKO-UKA and conventional methods yielded comparable scores for Visual Analogue Scale(VAS, *P* = 0.46 ), Pain Catastrophizing Scale(PCS, *P* = 0.3), American Knee Society Score (AKSS, *P* = 0.63), AKSS Total (*P* = 0.7), AKSS function(AKSSF, *P* = 0.66), Oxford Knee Score (OKS, *P* = 0.59); Forgotten Joint Score (FJS, *P* = 0.44), Mechanical femorotibial axis (MFTA, *P* = 0.07), Tibial component Coronal alignment (TCCA, *P* = 0.45), Tibial component posterior tilt(TCPT, *P* = 0.64), Lateral femoral component flexion-extension angle (LFCFEA, *P* = 0.96), Range of motion(ROM, *P* = 0.29 ), Complication rate(*P* = 0.53), Periprosthetic joint infection rate(PJI, *P* = 0.07), Revision rate(*P* = 0.14). However, MAKO-UKA was associated with improved femoral component coronal alignment (FCCA, *P* = 0.04), fewer FCCA outliers (*P* < 0.001), fewer TCCA outliers (*P* = 0.03), and reduced tibial posterior slope (PTS, *P* = 0.017), albeit with longer operative time (*P* < 0.001). Conclusion: While MAKO-UKA offers enhanced surgical precision and fewer alignment outliers, these technical advantages do not yet translate into superior short-term functional outcomes. Given the significant increase in procedural duration and the near-significant trend in infection rates, clinicians should remain vigilant. We emphasize that rigorous training and a focus on minimizing operative time are crucial for the safe implementation of this technology.

**Supplementary Information:**

The online version contains supplementary material available at 10.1007/s11701-026-03259-y.

## Introduction

To address long-standing challenges in component placement precision, robotic-assisted platforms for UKA were developed. These systems aim to optimize joint kinematics and potentially extend prosthesis survival by minimizing human error in bone resection and alignment [1]. However, as with any emerging technology, questions remain regarding its reliability in consistently achieving these goals. Previous meta-analyses comparing robotic-assisted UKA and conventional UKA (C-UKA) have reported heterogeneous findings due to the inclusion of different robotic systems [2–6]. Because each robotic platform differs in technical functionality and prosthesis integration, its clinical performance should be evaluated independently.

Among these systems, the Mako SmartRobotics™ platform has gained increasing clinical adoption over the past decade for its ability to enhance the precision of bone preparation and improve component alignment in UKA [7]. Despite its widespread use, few high-quality meta-analyses have focused specifically on the Mako system. To date, only one meta-analysis has directly compared the radiological and clinical outcomes of Mako-assisted UKA (MAKO-UKA) and C- UKA; however, that study included literature published only up to November 2021 and was restricted to English-language publications [8]. Consequently, its findings may not adequately represent current surgical practices or reflect data from non-English regions where the use of MAKO-UKA has grown substantially in recent years. Therefore, updated and more comprehensive evidence is warranted to capture contemporary global experience. We therefore performed this quantitative synthesis to consolidate the latest evidence comparing the surgical efficacy and safety profile of the MAKO platform against manual UKA procedures.

The aims of this systematic review and meta-analysis were: (i) to evaluate pain outcomes including Visual Analogue Scale (VAS) and Pain Catastrophizing Scale (PCS) following MAKO-UKA compared with C-UKA; (ii) to assess differences in functional outcomes including American Knee Society Score (AKSS), AKSS Total, AKSS function (AKSSF), Oxford Knee Score (OKS), and Forgotten Joint Score (FJS) between MAKO-UKA and C-UKA; (iii) to assess differences in radiological implant positioning and alignment accuracy including Mechanical femorotibial axis (MFTA), Femoral component Coronal alignment (FCCA), Tibial component Coronal alignment (TCCA), Tibial component posterior tilt(TCPT), Lateral femoral component flexion-extension angle (LFCFEA), FCCA outliers, and TCCA outliers between the two techniques; and (iv) to analyze perioperative metrics, including operative time, range of motion(ROM), Complication rate, Periprosthetic joint infection (PJI) rate, and revision rate.

## Methods

Prior to its commencement, the protocol for this systematic review was formally documented in the PROSPERO database (ID: CRD420250632120; available at: https://www.crd.york.ac.uk/PROSPERO/view/CRD420250632120), following the PRISMA guidelines to ensure transparency and rigor [9]. Definitions for radiological outcomes, including MFTA, FCCA, TCCA, TCPT, and LFCFEA, are provided in Table 1.

**Table 1:** Operational definitions of radiographic metrics

Outcome	Definition
**FCCA**	The Femoral component Coronal alignment (FCCA) angle is measured as the acute angle between the femoral component and the femoral diaphyseal axis in the coronal plane on the screened short leg X-rays
**TCCA**	Tibial component Coronal alignment (TCCA)angle is measured as the acute angle between a line perpendicular to the tibial axis and a line drawn across the tibial tray in the coronal plane on a short leg screened X-ray
**MFTA**	The Mechanical femorotibial angle (MFTA) is the angle subtended by a line extending from the center of the femoral head to the center of the knee joint to the center of the ankle mortise.
**TCPT**	The Tibial component posterior tilt (TCPT)is measured as the acute angle between a line drawn along the tibial tray and a line perpendicular to the tibial axis in the lateral short leg screened view
**LFCFEA**	The Lateral femoral component flexion-extension angle (LFCFEA) is measured as the acute angle between a line through the center of the femoral peg and the femoral axis in the lateral short leg screened view in UKA with Zimmer Biomet Oxford partial knee. However, in the Restoris^®^ MCK UKA, there is a 30° angle between the femoral condyle axis and the femoral peg, which was subtracted from the measured angle while evaluating the femoral component flexion/extension on Restoris^®^ MCK UKA

Operational definitions of radiographic metrics: MFTA, FCCA, TCCA, TCPT, and LFCFEA

FCCA: Femoral component Coronal alignment; TCCA: Tibial component Sagittal alignment; MFTA: mechanical femorotibial axis; TCPT: Tibial component posterior tilt; LFCFEA: Lateral femoral component flexion-extension angle

### Search strategy

A comprehensive search of the PubMed, Web of Science, Cochrane Library, Embase, Scopus, ClinicalTrials.gov, China National Knowledge Infrastructure (CNKI), Wanfang, China Biology Medicine Disc (CBM), and China Science and Technology Journal (CSTD) databases was performed to identify comparative studies of MAKO-UKA and C-UKA published before October 2025. The detailed search algorithms for each database, including Boolean operators and search terms, are provided in Supplementary Data 1.

Two independent reviewers (X.J.Z and Q.M.) screened all titles, abstracts, and full texts to identify eligible studies. Discrepancies were resolved through consultation with a third senior investigator (C.J.S). Inter-rater reliability was evaluated using Cohen’s κ coefficient.

### Inclusion and exclusion criteria

Studies qualified for inclusion if they utilized a comparative design (such as RCTs or observational cohorts) focusing on patients with end-stage knee osteoarthritis treated with either robotic or manual UKA. Eligibility further required the assessment of at least one clinical or radiographic parameter, including VAS, PCS, AKSS, AKSS Total, AKSSF, OKS, FJS, MFTA, FCCA, TCCA, TCPT, LFCFEA, FCCA outlier, TCCA outlier, operation time, ROM, Complication rate, PJI rate, and revision rate. Furthermore, we only considered studies providing enough raw data to derive effect sizes, specifically risk ratios (RR), odds ratios (OR), or mean differences (MD). Conversely, non-primary research—such as narrative reviews, editorials, and case series—along with conference abstracts and any publications lacking extractable quantitative results, were strictly omitted from this analysis.

### Data extraction process

A pair of independent investigators (X.J.Z and Q.M.) performed the data harvesting. In instances of disagreement, a senior author (C.J.S.)was consulted to reach a consensus. The information harvested from each trial spanned three primary domains: trial-specific descriptors (including authorship, publication year, geographic origin, and methodology), patient-level baseline attributes (notably age, gender distribution, body mass index, and monitoring duration), and all pre-specified clinical outcomes. To maintain the integrity of our dataset, we proactively reached out to the primary investigators via electronic correspondence whenever essential data points were absent or required further explanation.

### Data transformation

For trials that reported medians with ranges or interquartile ranges, we reconstructed the corresponding means and standard deviations (SDs) to ensure data consistency. This approximation was based on the mathematical frameworks validated by Luo et al. [9] and Wan et al. [9]. Such imputation techniques are well documented in the literature [10–13]. Such imputation techniques are well documented in the literature.

### Quality assessment

The methodological rigor of the included evidence was assessed using specialized instruments tailored to the study design. Specifically, the Newcastle–Ottawa Scale (NOS) [14] was applied to observational cohorts, with a particular emphasis on the integrity of patient selection, the adequacy of comparability controls, and the reliability of outcome tracking. Concurrently, the risk of bias in randomized controlled trials (RCTs) was assessed using the standard frameworks documented in the Cochrane Handbook. This critical appraisal was conducted in parallel by two researchers (X.J.Z. and Q.M.). Any divergent assessments of study quality were reconciled through consultation with a senior investigator (C.J.S.) until consensus was reached.

### Data analyses

Statistical computations were performed using Stata (version 18.0; StataCorp). Continuous data were presented as mean ± SD. We quantified inter-study inconsistency using the $\:{I}^{2}$ index, while the Cochran Q test was employed to identify significant heterogeneity. Given anticipated clinical and methodological diversity, all analyses employed a random-effects model using the Restricted Maximum Likelihood (REML) method [15].

Effect sizes were expressed with 95% confidence intervals (CIs). Dichotomous variables (e.g., FCCA outliers and TCCA outliers) were analysed using odds ratios (ORs), which approximate relative risk (RR) under Cornfield’s rare-outcome assumption [16]. Continuous outcomes (VAS, PCS, AKSS, AKSS Total, AKSSF, OKS, FJS, MFTA, FCCA, TCCA, TCPT, LFCFEA, operative time, and ROM) were assessed using mean differences (MDs). Statistical significance was defined by a two-tailed P-value threshold of 0.05. To detect potential publication bias, we applied Egger’s linear regression test to any outcome synthesized from eight or more independent datasets, with a P-value below 0.10 serving as the diagnostic cutoff for significant asymmetry. Furthermore, the stability of our meta-analytic results was verified through leave-one-out sensitivity procedures; by iteratively excluding one study at a time, we ensured that the overall pooled estimates remained resilient and were not disproportionately influenced by any single trial.

### Search results

**Fig. 1:**
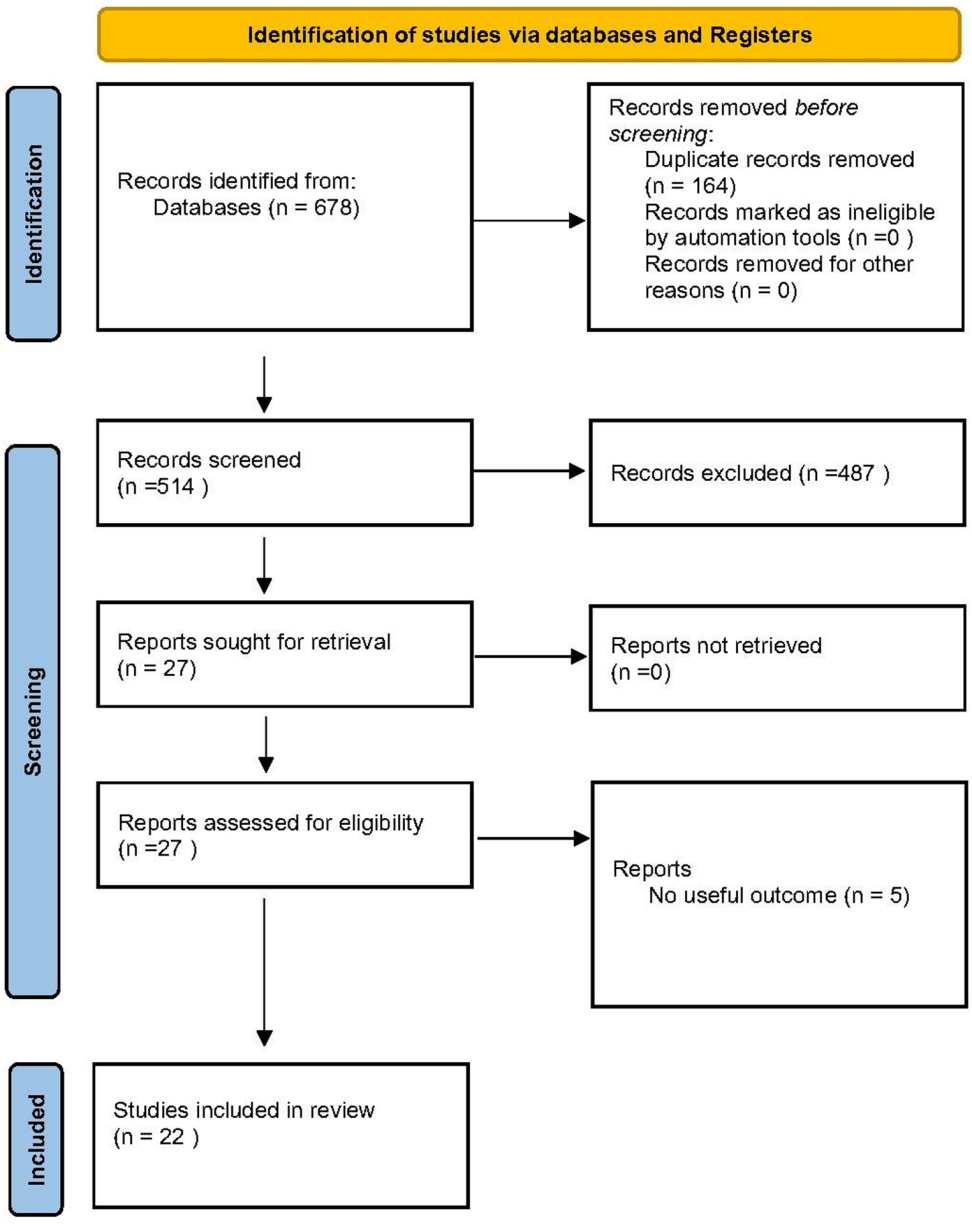
Flowchart depicting the identification, screening, and inclusion of eligible literature

The PRISMA flow diagram summarizes the study selection process. (Fig. 1) The complete search strategy used for each database is provided in Supplementary Data 1. The initial database search yielded 678 records. After importing these records into Zotero, 164 duplicate records were removed. Of the remaining 514 unique articles, 487 were excluded after review of titles and abstracts for irrelevant populations, interventions, study types, or outcomes. The remaining 27 full-text articles were assessed, and 5 were excluded for insufficient or inconsistent outcome data. Ultimately, 22 studies [17–38] met the inclusion criteria. Inter-reviewer agreement was excellent (κ = 0.9040 for title/abstract screening; κ = 0.8670 for full-text review) (see Supplement data 2).

### Sample characteristics

Tables 2 and 3 provide a detailed overview of the baseline attributes and clinical outcomes collected from each trial. The literature identified for this systematic review was published between 2010 and 2025.

**Table 2:** Baseline profiles and descriptive attributes of included trials

The detailed baseline characteristics information								
Author/year	Country	Study type	MAKO-UKA/C-UKA					
			Patients	UKAs	Mean age(years)	Womenr(%)	BMI	Follow-up time(month)
**Banger 2021**	United Kingdom	RCT	55/49	55/49	NA	NA	NA	60
**Bell 2016**	United Kingdom	RCT	70/69	70/69	62.5/61.7	54/56	NA	NA
**Blyth 2017**	United Kingdom	RCT	64/65	64/65	NA	NA	NA	12
**Çabuk 2022**	Türkiye	RCS	36/57	36/57	60.9/62.9	81/86	NA	12
**Clement 2023**	United Kingdom	RCT	64/65	64/65	62.1/62.5	44/46	NA	60
**Cool 2019**	United States	RCS	246/492	246/492	NA	53.66/57.32	NA	24
**FuJun 2017**	China	RCS	10/36	15/45	64.5/63.7	90/70	27.8/27	3
**Gilmour 2018**	United Kingdom	RCT	58/54	58/54	61.8/62.6	44.8/48.1	NA	24
**Hansen 2014**	United States	RCS	30/32	30/32	57.13/60.66	46.7/65.6	32.13/33.34	24
**Katherine 2016**	United States	RCS	87/177	87/177	NA	NA	NA	32
**Kayani 2018**	United Kingdom	PCS	60/60	60/60	64.1/65.5	26/27	28.1/27.3	NA
**Kayani 2019**	United Kingdom	PCS	73/73	73/73	65.3/66.1	41/39	28.7/27.9	NA
**Lonner 2010**	United States	PCS	31/27	31/27	64/57	51.6/37	30/28	3
**Maritan 2023**	Italy	RCS	52/43	52/43	60.9/61.5	78.8/86	26.2/27	95
**Motesharei 2018**	United Kingdom	RCT	31/39	31/39	62.7/64.6	38.7/38.5	33.86/30.85	12
**Park 2019**	Korea	RCS	55/57	55/57	64.8/68.4	80/87.7	25.5/25.9	27.8
**St Mart 2020**	Australia	RCS	2851/3093	2851/3093	65.7/65.4	43.6/41.1	NA	36
**Tan 2025**	Singapore	RCS	49/111	49/111	63.5/63.2	69.4/57.3	27.6/28.1	3
**Thilak 2020**	India	RCS	24/12	24/12	57.5/53.7	75/83.3	NA	1
**Wong 2019**	United States	RCS	58/118	58/118	70.4/67.9	49/63	28.2/28.7	3
**Wu 2021**	China	RCS	52/61	52/61	68.5/69.4	78.8/85.2	NA	24
**Yin 2021**	China	RCT	39/39	39/39	71.6/72.1	48.7/43.6		6

Baseline profiles and descriptive attributes of included trials, encompassing study design, geographic origin, and cohort demographics

BMI: body mass index; UKA: unicompartmental knee arthroplasty; RCT: Randomized control trial; RCS: Retrospective cohort study; PCS: Prospective cohort study; MAKO-UKA: MAKO-assisted unicompartmental knee arthroplasty; C-UKA: Conventional unicompartmental knee arthroplasty

**Table 3:** Comprehensive data reporting for all synthesized clinical and radiographic endpoints

Author/year	Outcome
**Banger 2021**	OKS, AKSS Objective, AKSS Function, AKSS Total, FJS, PCS, VAS, stiffness VAS, ROM, VAS, Complication rate, revision rate, PJI rate
**Bell 2016**	Outlier of FCCA, Outlier of TCCA
**Blyth 2017**	VAS, AKSS, OKS, FJS, SF-12 physical, SF-12 mental, complication rate, revision rate, PJI rate
**Çabuk 2022**	FCCA, TCCA, LFCFEA, TCPT
**Clement 2023**	Revision rate
**Cool 2019**	Revision rate
**FuJun 2017**	Operation time, blood loss, FCCA, TCCA, TCPT, LFCFEA, revision rate
**Gilmour 2018**	AKSS, OKS, FJS, VAS, Stiffness VAS, PCS, ROM, revision rate
**Hansen 2014**	ROM, TCCA, TCPT, revision rate, PJI rate
**Katherine 2016**	Revision rate, TCCA༌TCPT, outliers of TCCA、TCPT, Operation time
**Kayani 2018**	Operation time, Complication rate
**Kayani 2019**	VAS pian, ROM, MFTA, Complication rate
**Lonner 2010**	TCCA, complication rate
**Maritan 2023**	Revision rate
**Motesharei 2018**	Revision rate
**Park 2019**	MFTA, FCCA, Outliers of FCCA, TCCA, outliers of TCCA, TCPT, ROM, AKSS Objective, AKSS function, WOMAC (total), WOMAC stiffness, WOMAC Pain༌WOMAC Function
**St Mart 2020**	Revision rates, PJI rate
**Tan 2025**	Operation time, OKS, VAS, AKSS Objective, ROM, Complication rate
**Thilak 2020**	MFTA, TCPT
**Wong 2019**	SF-12 physical, SF-12 mental༌WOMAC pain༌WOMAC stiffness༌WOMAC function༌AKSS Function༌revision rate, PJI rate, Operation time
**Wu 2021**	TCCA, TCPT, Operation time༌blood loss, outlier of TCCA, Complications༌VAS, WOMAC total
**Yin 2021**	Operation time, blood loss༌VAS, AKSS objective, AKSS function, complication rate

Comprehensive data reporting for all synthesized clinical and radiographic endpoints

OKS, Oxford Knee Score; AKSS, American Knee Society Score; FJS, Forgotten Joint score; PCS, Pain Catastrophizing Scale; VAS, Visual analog scale; ROM, range of motion; PJI, periprosthetic joint infection; FCCA, Femoral component Coronal alignment; TCCA, Tibial component Sagittal alignment; SF-12, 12-Item Short Form Health Survey; LFCFEA, Lateral femoral component flexion-extension angle; TCPT, Tibial component posterior tilt; MFTA, mechanical femorotibial axis

## Results

### Quality assessment

Fifteen non-randomized studies achieved NOS scores of 7–8 (out of 9), indicating moderate-to-good quality (Table 4). Common limitations included incomplete follow-up and potential outcome assessment bias, which are inherent to observational designs.

**Table 4:** Quality appraisal scores for the non-randomized evidence

Risk-of-bias assessment for the studies included in the meta-analysis (NOS)									
(nRCT) Study = 15	Selection				Comparability	Outcome/Exposure			Score
	Item 1	Item 2	Item 3	Item 4	Item 5	Item 6	Item 7	Item 8	
**Çabuk 2022**	*		*	*	**	*		*	**7**
**Cool 2019**	*		*	*	**	*	*	*	**8**
**FuJun 2017**	*		*	*	**	*		*	**7**
**Hansen 2014**	*		*	*	**	*	*	*	**8**
**Katherine 2016**	*		*	*	**	*	*	*	**8**
**Kayani 2018**	*		*	*	**	*		*	**7**
**Kayani 2019**	*		*	*	**	*		*	**7**
**Lonner 2010**	*		*	*	**	*		*	**7**
**Maritan 2023**	*		*	*	**	*	*	*	**8**
**Park 2019**	*		*	*	**	*		*	**7**
**St Mart 2020**	*		*	*	**	*	*	*	**8**
**Tan 2025**	*		*	*	**	*		*	**7**
**Thilak 2020**	*		*	*	**	*		*	**7**
**Wong 2019**	*		*	*	**	*		*	**7**
**Wu 2021**	*		*	*	**	*	*	*	**8**

Quality appraisal scores for the non-randomized evidence, demonstrating scores between 7 and 8

Item 1, Is the case definition adequate / Representativeness of the exposed cohort

Item 2, Representativeness of the case / Selection of the non-exposed cohort

Item 3, Selection of controls / Ascertainment of exposure to implants

Item 4, Definition of controls / Demonstration that outcome of interest was not present at start of study

Item 5, Comparability of cases and controls on the basis of design or analysis / Comparability of cohorts on the basis of the design or analysis

Item 6, Ascertainment of exposure / Assessment of outcome

Item 7, Same method of ascertainment for cases and controls / Was follow up long enough for outcomes to occur

Item 8, Non-response rate / Adequacy of follow up of cohorts

Our quality appraisal of the seven RCTs, conducted via the Cochrane risk-of-bias instrument, is summarized in Table 5. The methodological integrity of these trials was generally satisfactory, particularly regarding the robust execution of randomization and the concealment of treatment allocation. Nonetheless, performance and detection bias (specifically the blinding of subjects and surgical staff) were consistently rated as high risk; this is an inherent limitation in orthopedic research, where the physical nature of robotic versus manual procedures precludes effective masking.

**Table 5:** Risk of bias profiling for randomized controlled trials

Methodological Assessment According to Six Domains of Potential Biases (Cochrane Risk of Bias Tool)							
RCT Study = 7	Random Sequence Generation	Allocation Concealment	Blinding of Participants and Personnel	Blinding of Outcome Assessment	Incomplete Outcome Data	Selective Reporting	Other Bias
**Banger 2021**	Low	Low	High	Low	Low	Low	Unclear
**Bell 2016**	Low	Low	High	Low	Low	Low	Unclear
**Blyth 2017**	Low	Low	High	Low	Low	Low	Unclear
**Clement 2023**	Low	Low	High	Low	Low	Low	Unclear
**Gilmour 2018**	Low	Low	High	Low	Low	Low	Unclear
**Motesharei 2018**	Low	Low	High	Low	Low	Low	Unclear
**Yin 2021**	Low	Low	High	Low	Low	Low	Unclear

Risk of bias profiling for randomized controlled trials utilizing the Cochrane standardized framework

RCT, Randomized controlled trials. The RCTs’ methodological quality and basis were assessed as follows: randomization, allocation concealment, blind method, selective reporting, group similarity at baseline, incomplete outcome data, compliance, the timing of outcome assessments, and intention-to-treat analysis

### Publication bias assessment

In instances where outcomes were supported by at least eight independent datasets, we utilized Egger’s regression to screen for potential publication bias. Our analysis indicated a lack of significant asymmetry for TCCA (*P* = 0.5403), complication incidence (*P* = 0.7244), and revision requirements (*P* = 0.6293), suggesting that the pooled results for these parameters were not substantially influenced by small-study effects. (see Supplementary Data 3).

### Sensitivity analysis

Leave-one-out analyses demonstrated that pooled estimates remained largely stable, confirming the robustness of the results. However, the PJI rate showed sensitivity to individual study exclusion (see Supplementary Data 4).

### Outcome measurement

#### Pain

Pain administered across these studies included Pain VAS(*n* = 7) and PCS(*n* = 2). Statistical analysis revealed that the robotic group did not significantly outperform the manual group in subjective pain relief with Pain VAS (MD = -2.11, 95% CI [-7.68, 3.46], *P* = 0.46, Fig. 2.1) and PCS (MD = 0.68, 95% CI [-0.6, 1.96], *P* = 0.3, Fig. 2.2).

**Fig. 2:**
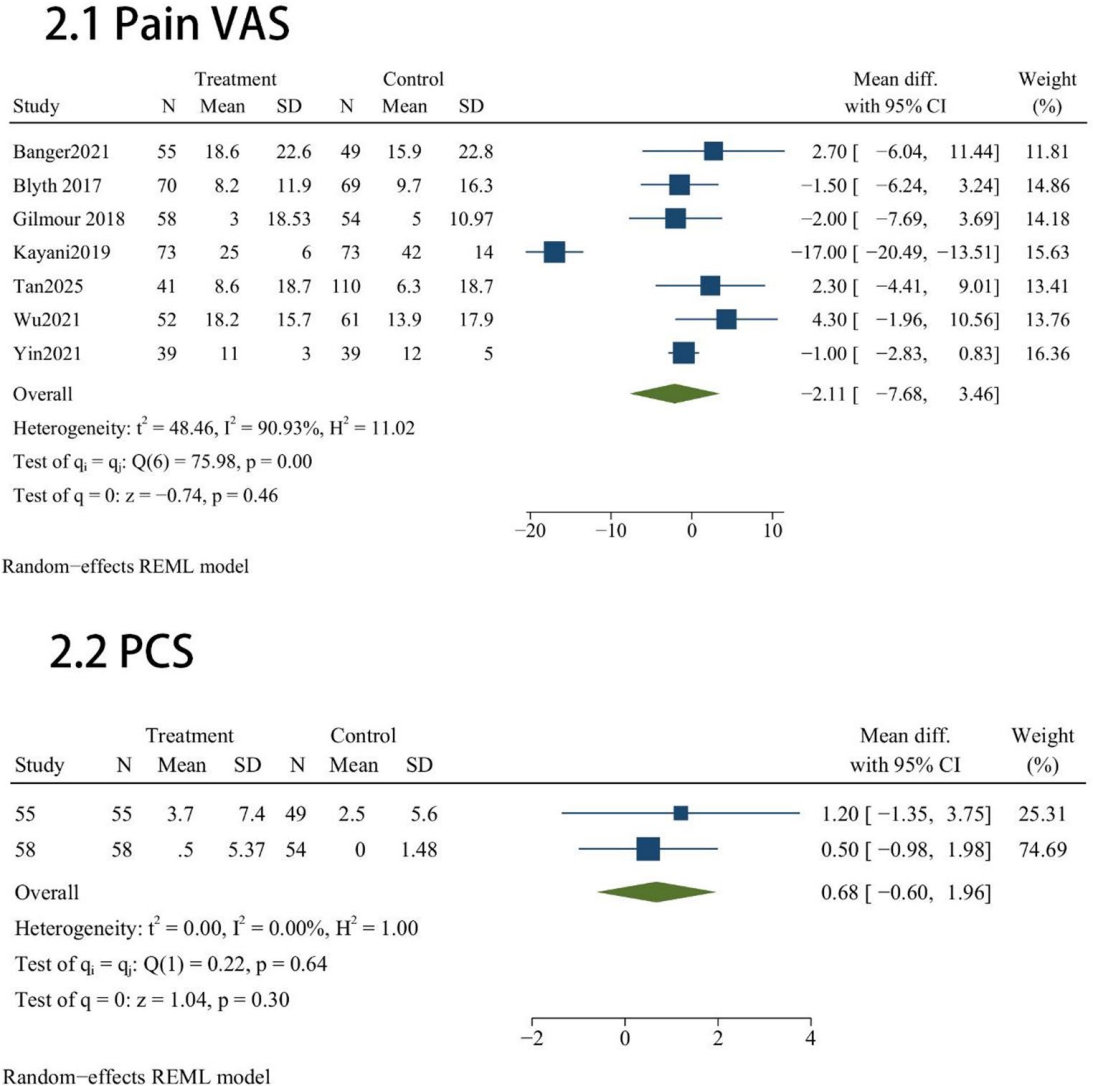
Quantitative synthesis of pain relief: Forest plots for VAS and PCS

### Function outcome results

Function scales administered across these studies included AKSS total(*n* = 3), AKSS(*n* = 4), AKSS function(*n* = 4), OKSS(*n* = 4) and FJS(*n* = 3). Regarding functional recovery, our comparative analysis indicated that the robotic cohort did not achieve superior outcomes over the manual group across any of the assessed scales. Statistically equivalent scores were observed for AKSS total (MD = -1.42; 95% CI, -8.54 to 5.7; *P* = 0.7; Fig. 3.1), individual AKSS (MD = 1.72; 95% CI, -5.29 to 8.73; *P* = 0.63; Fig. 3.2), and AKSS function (MD = 1.53; 95% CI, -5.36 to 8.41; *P* = 0.66; Fig. 3.3). Similarly, patient-reported functionality showed no meaningful disparities between the two techniques, as evidenced by the pooled results for OKS (MD = 0.28; 95% CI, -0.74 to 1.3; *P* = 0.59; Fig. 3.4) and FJS (MD = 3.17; 95% CI, -4.8 to 11.14; *P* = 0.44; Fig. 3.5).

**Fig. 3:**
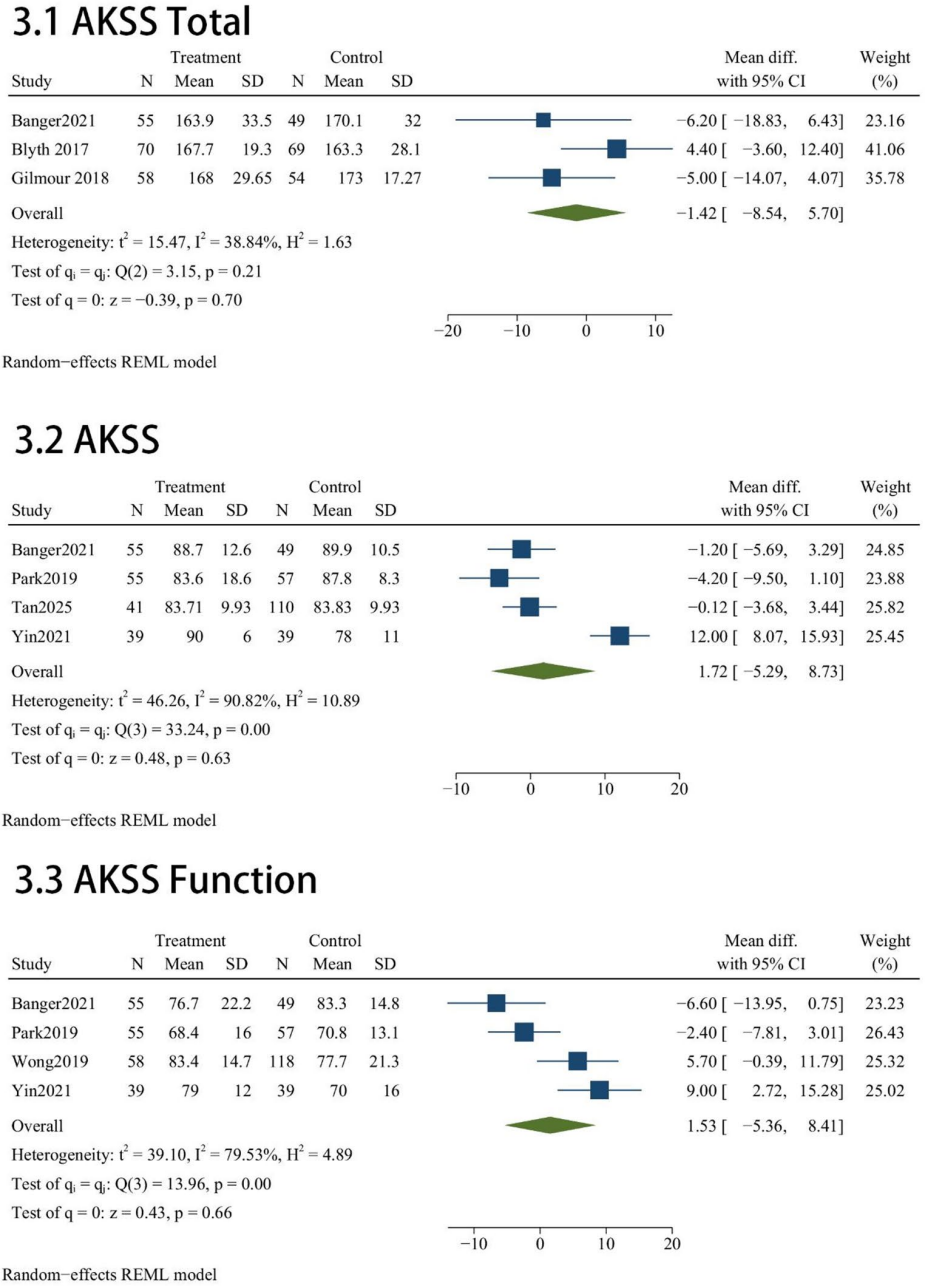

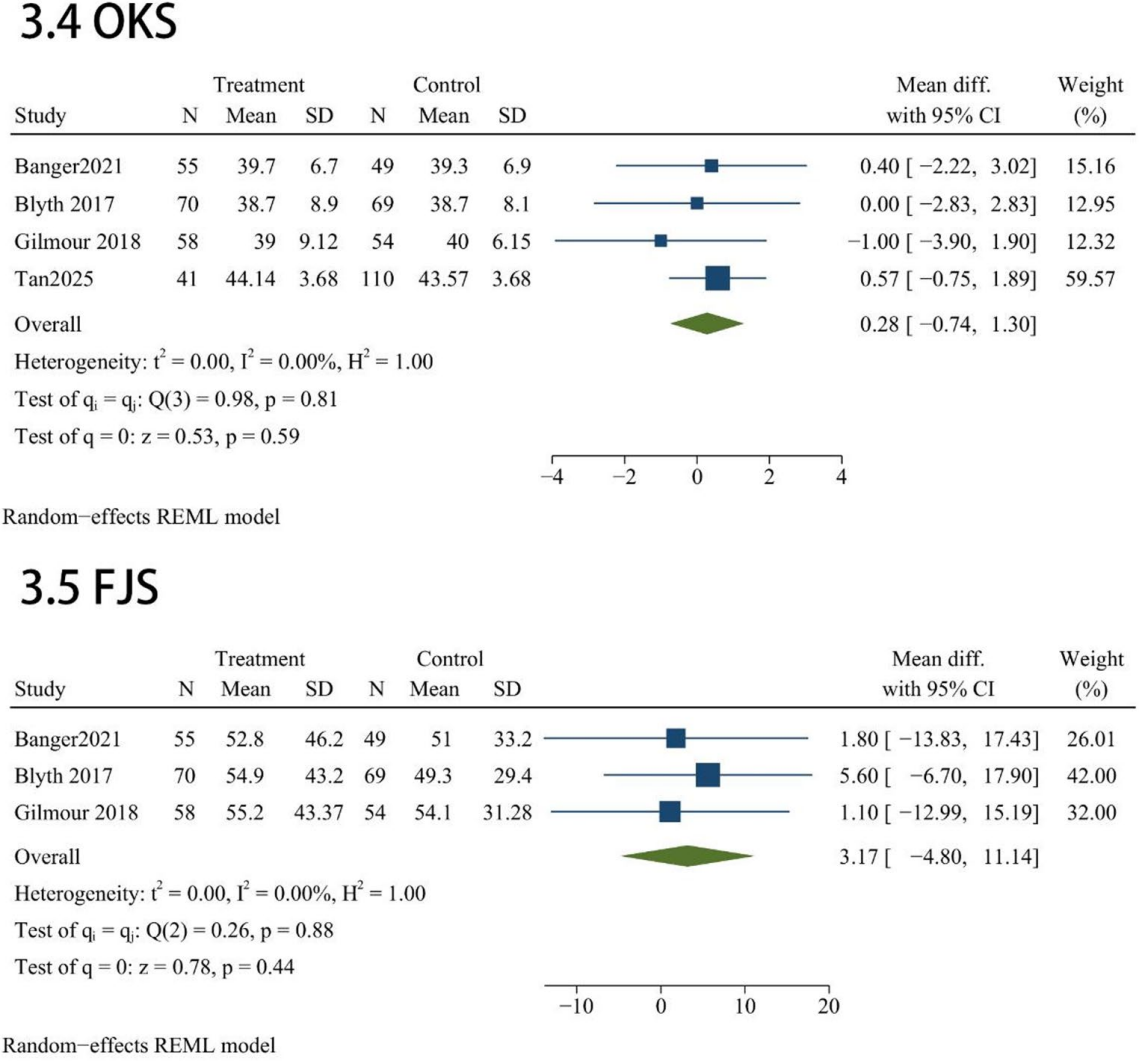
Forest plots evaluating functional recovery through AKSS, OKS, and FJS metrics

### Radiological implant position and alignment

Radiological Implant Position and Alignment results administered across these studies included MFTA (*n* = 3), FCCA (*n* = 3), TCCA (*n* = 8), TCPT (*n* = 7), LFCFEA(*n* = 2), FCCA outlier (*n* = 2), TCCA outlier (*n* = 4). Radiographic comparisons indicated that MAKO-UKA and C-UKA yielded comparable results across several parameters, with no statistically significant disparities observed for MFTA (MD = -0.64; 95% CI, -1.33 to 0.05; *P* = 0.07; Fig. 4.1), TCCA (MD = -0.45; 95% CI, -1.62 to 0.72; *P* = 0.45; Fig. 4.3), TCPT (MD = -0.35; 95% CI, -1.83 to 1.13; *P* = 0.64; Fig. 4.4), or LFCFEA (MD = 0.07; 95% CI, -2.87 to 3.01; *P* = 0.96; Fig. 4.5). However, robotic assistance demonstrated a advantage in optimizing alignment precision. Specifically, the MAKO-UKA cohort achieved a significantly lower FCCA (MD = -1.17; 95% CI, -2.26 to -0.07; *P* = 0.04; Fig. 4.2). Furthermore, the use of the robotic platform was associated with a marked improvement in surgical consistency, as evidenced by a substantial reduction in the risk of outliers for both FCCA (OR = 0.21; 95% CI, 0.096 to 0.460; *P* < 0.001; Fig. 4.6) and TCCA (OR = 0.387; 95% CI, 0.166 to 0.901; *P* = 0.03; Fig. 4.7) compared to the manual group.

**Fig. 4:**
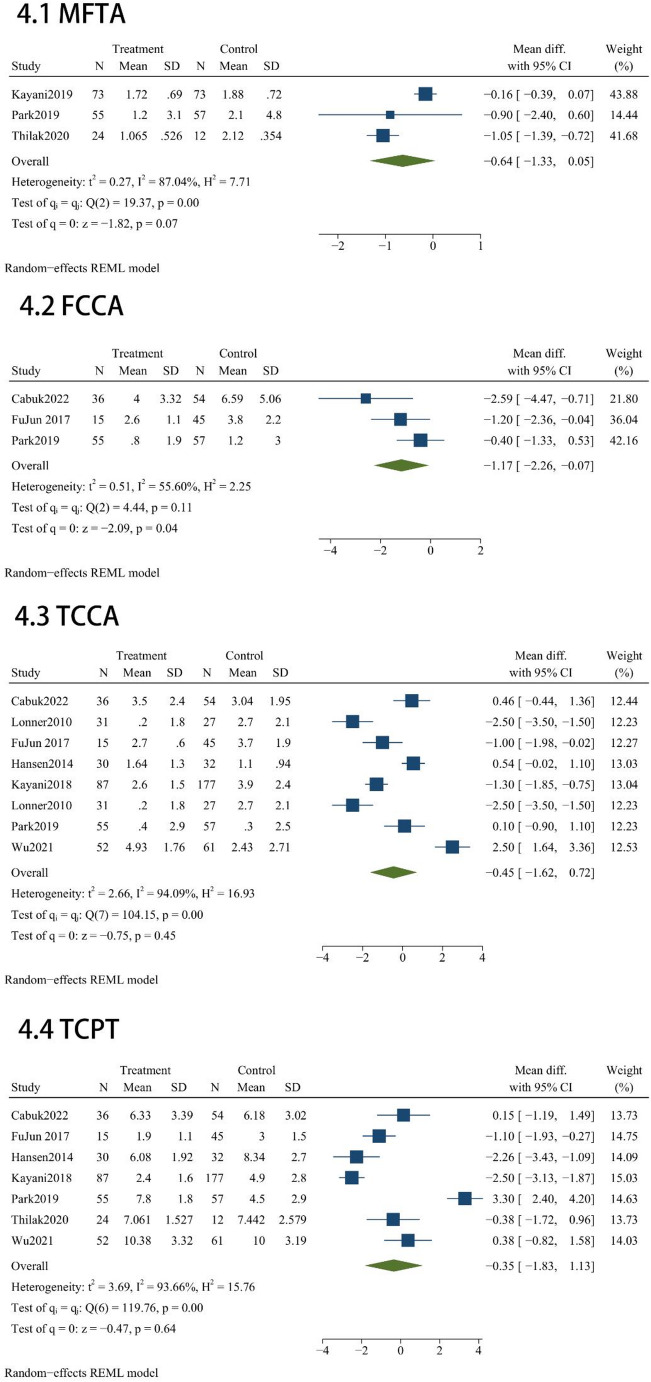

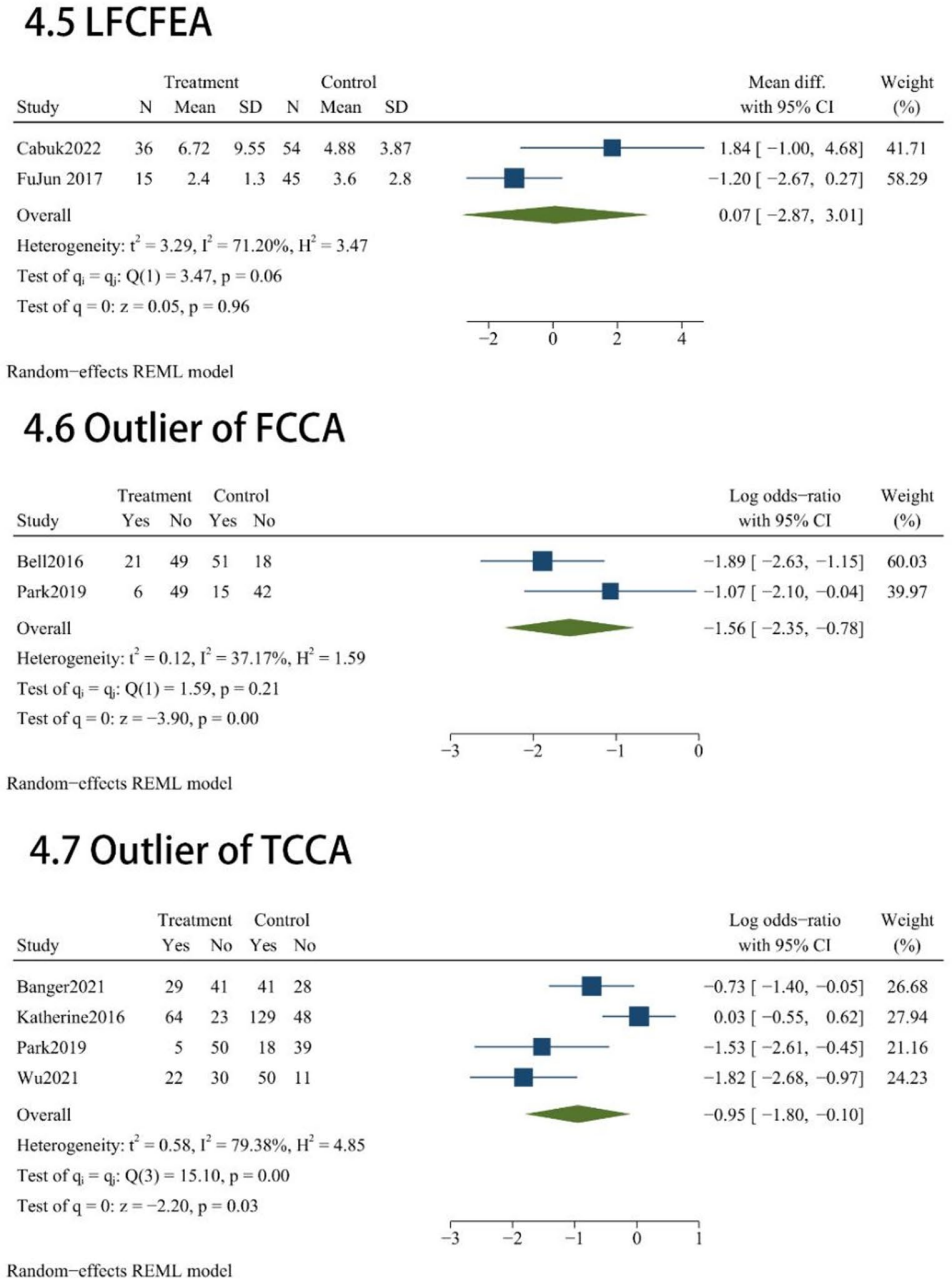
Radiographic accuracy assessment: Forest plots for coronal/sagittal alignment and outlier incidences

### Perioperative metrics results

In terms of perioperative performance and safety, MAKO-UKA and C-UKA exhibited comparable results across several key indicators. No statistically significant disparities were observed in range of motion (ROM) (MD = 2.54; 95% CI, -2.13 to 7.2; *P* = 0.29; Fig. 5.1) or overall safety profiles. Specifically, the two techniques yielded similar risks for general complications (RR = 0.718; 95% CI, 0.258 to 2.004; *P* = 0.53; Fig. 5.3), prosthetic joint infection (PJI) (RR = 2.1; 95% CI, 0.95 to 4.57; *P* = 0.07; Fig. 5.4), and surgical revision (RR = 0.67; 95% CI, 0.39 to 1.14; *P* = 0.14; Fig. 5.5). However, robotic assistance was associated with a significant increase in procedural duration; the pooled analysis indicated that the MAKO-UKA group required an average of 19.69 min longer to complete than the manual cohort (MD = 19.69; 95% CI, 11.75 to 27.63; *P* < 0.001; Fig. 5.2).

**Fig. 5:**
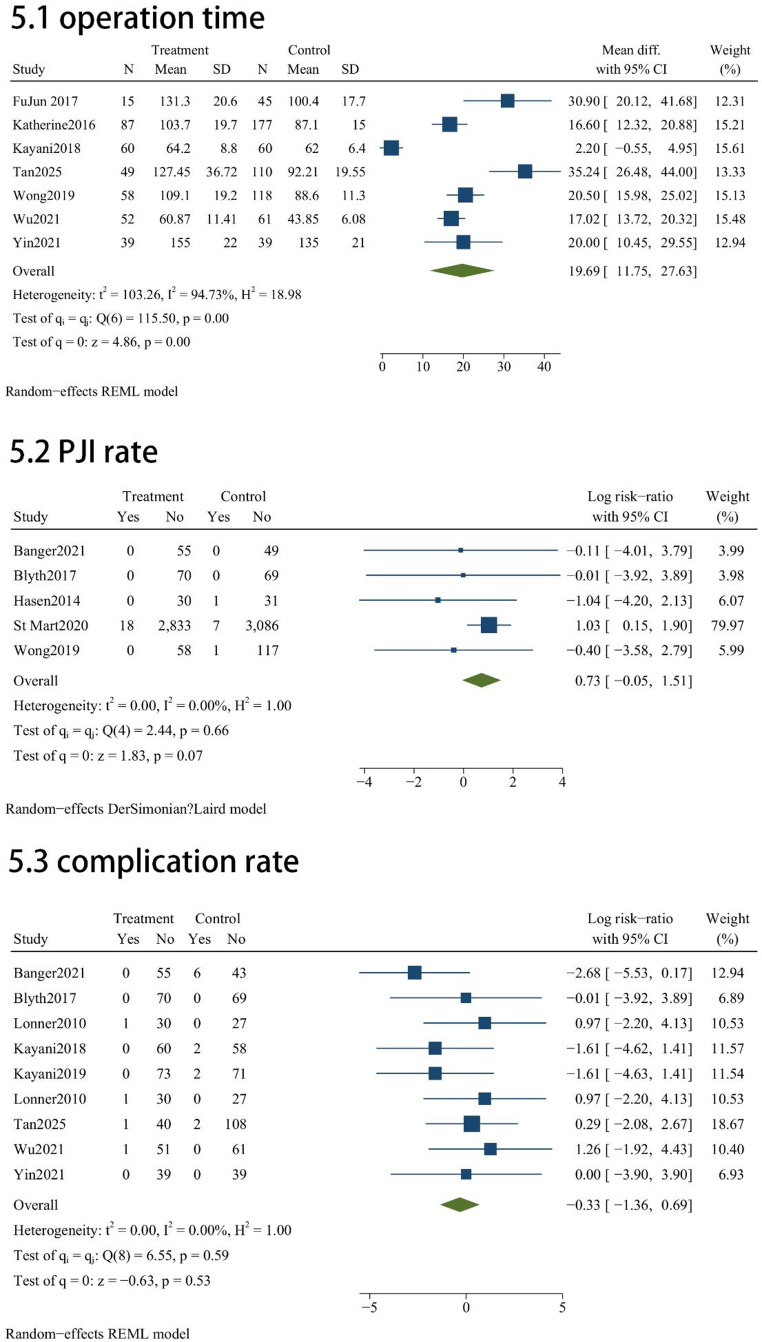

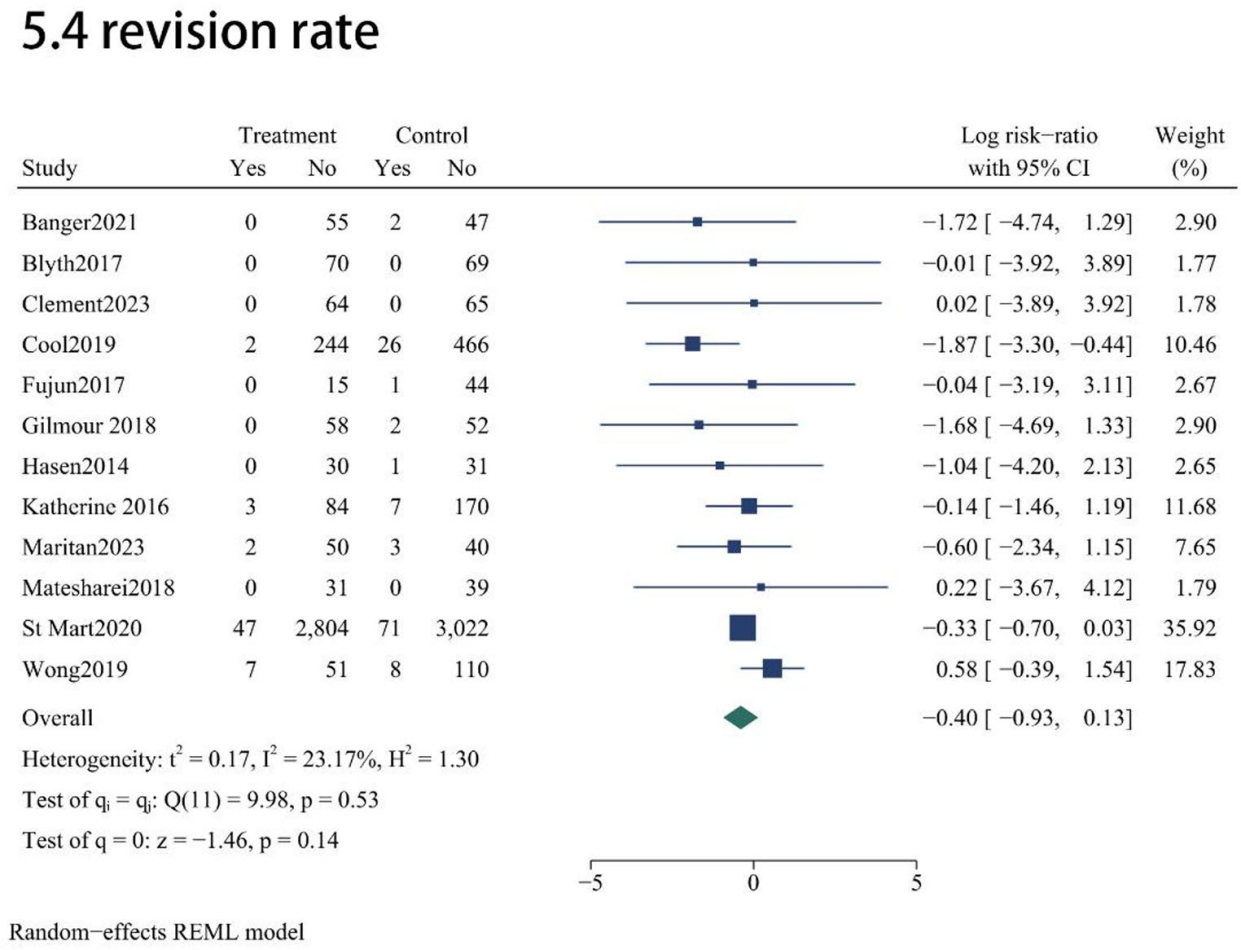
Forest plots for operation time and postoperative safety profiles (PJI, overall complications, and revisions)

## Discussion

By isolating a single robotic platform, this meta-analysis minimizes heterogeneity arising from differing robotic technologies [39–43] and provides a more precise assessment of MAKO-UKA compared with C-UKA.

The principal finding of this analysis is that MAKO-UKA achieves significantly improved radiographic alignment and fewer component outliers, yet these advantages do not appear to translate into superior short- to mid-term clinical outcomes. This discrepancy highlights the ongoing debate over whether enhanced mechanical precision necessarily yields improved functional outcomes [25, 26]. The MAKO system’s CT-based preoperative planning and intraoperative haptic feedback clearly improve the accuracy and reproducibility of bone resections and implant positioning [44–46]. However, patient-reported outcomes are multifactorial and influenced not only by alignment but also by soft-tissue balance, rehabilitation quality, and patient expectation [27]. As such, radiological precision alone may not be sufficient to yield noticeable clinical benefit in the early postoperative period. Consequently, technical excellence in radiographic accuracy does not immediately translate into superior functional performance in the early stages of recovery. Extended longitudinal monitoring is therefore essential to ascertain whether these alignment improvements ultimately bolster prosthesis longevity or mitigate the risk of late-stage complications.

From a clinical perspective, the improved alignment achieved with MAKO-UKA is not trivial. Biomechanical evidence underscores that even a 3° positioning error can escalate strain at the tibial baseplate-cement junction by 40% [47, 48], potentially predisposing to early loosening. Therefore, robotic assistance may enhance implant durability and long-term stability. Nevertheless, the absence of a short-term functional benefit in this analysis suggests that early clinical metrics may not fully capture the potential long-term advantages of improved alignment.

A consistent limitation of robotic-assisted procedures remains the longer operative time compared with conventional UKA [49–51]. This finding was reaffirmed in the present analysis. The additional time reflects both the technical setup—such as registration, calibration, and intraoperative verification—and the inherent learning curve associated with robotic workflows. While this initially prolongs surgery, multiple reports have demonstrated that operative efficiency improves substantially with increasing surgeon experience [52]. As robotic technology continues to evolve with faster registration protocols and improved software interfaces, this time differential may become less clinically significant. Nonetheless, medical centers adopting MAKO systems should anticipate a structured learning period.

Regarding perioperative safety, the profiles of MAKO-UKA and C-UKA were similar overall and there was no statistically significant difference in rates of complications or revision. In theory, the MAKO system’s haptic limits and bone amputation under control could decrease iatrogenic soft-tissue damage and thermal tissue necrosis [36, 37], However, these potential biological advantages may be offset by the increased complexity of the robotic workflow, which added an average of 19.69 min to the procedural duration. While the difference in PJI rates did not reach the threshold for statistical significance (*P* = 0.07), this marginal increasing trend in PJI with the significantly prolonged operative time warrants clinical vigilance. Since prolonged surgical exposure is a well-established risk factor for infection, radiographic precision improvement must be carefully balanced against these perioperative factors. The learning curve associated with the MAKO system may contribute to these longer times. Structured surgical training and team-based proficiency are essential to mitigate these risks and improve the overall safety profile.

The economic implications of MAKO-UKA merit careful consideration. The Mako platform entails substantial capital investment, software licensing, maintenance, per-case consumable costs, and additional preoperative imaging [53–55]. Current economic analyses suggest that cost-effectiveness is highly volume-dependent. Busy surgical centers may neutralize procedural costs by optimizing clinical throughput and decreasing the long-term burdens of reoperation or adverse events [40, 41]. However, smaller centers may find it challenging to achieve financial sustainability unless clear long-term clinical benefits are demonstrated [56]. In this meta-analysis, no reduction in revision or complication rates was observed, underscoring the continued uncertainty regarding the cost-benefit balance. A comprehensive evaluation of long-term outcomes, including survivorship and cost-utility modeling across healthcare systems, will be essential for defining the true value of MAKO-UKA.

In summary, the evidence derived from this pooled analysis demonstrates that MAKO-UKA enhances surgical precision and radiographic accuracy without demonstrating short-term clinical superiority over C-UKA. These findings underscore the distinction between technical excellence and clinical efficacy. The potential long-term benefits of MAKO-UKA precision, including improved implant longevity and reduced complications, remain plausible but unproven. As robotic technology becomes more accessible, the balance between precision, efficiency, and economic feasibility will determine its ultimate role in modern arthroplasty practice.

### Potential limitations

Certain inherent constraints warrant consideration when weighing the conclusions of this synthesis.Despite an extensive literature search, the number of eligible studies was relatively small for certain endpoints, precluding meta-regression analysis and limiting exploration of sources of heterogeneity. Methodological differences in patient selection, surgical technique, and outcome assessment likely contributed to variability among studies. Moreover, the brevity of observation in most incorporated trials hampers a comprehensive appraisal of multi-year prosthesis survival and sustained functional outcomes. Surgeon-related factors, such as robotic experience and case volume, were also inconsistently reported, which may significantly influence both accuracy and outcomes. Future studies employing standardized methodologies, longer follow-up periods, and subgroup analyses that account for surgeon experience are warranted.

## Conclusion

In conclusion, while MAKO-UKA offers enhanced surgical precision and fewer alignment outliers, these technical advantages do not yet translate into superior short-term functional outcomes. Given the significant increase in procedural duration and the near-significant trend in infection rates, clinicians should remain vigilant. We emphasize that rigorous training and a focus on minimizing operative time are crucial for the safe implementation of this technology.

## Supplementary Information

Below is the link to the electronic supplementary material.

Supplementary Material 1

## Data Availability

The analytical datasets and harvesting templates underpinning these results are obtainable from the lead author via a justified inquiry.
