# Supplementary material for: Comparison of MAKO robotic-assisted and manual unicompartmental knee arthroplasty: a meta-analysis of radiographic precision and short-term functional results
Source: J Robot Surg. 2026 Mar 2;20(1):307. doi: 10.1007/s11701-026-03259-y (PMC12953483; doi:10.1007/s11701-026-03259-y)
Supplement: Supplementary file 1 — Supplementary Material 1 [file 11701_2026_3259_MOESM1_ESM.zip › Supplementary Appendix/data 1 Search strategy.docx]

| **Database** | **Search strategy(October 18, 2025 )** | **Number of articles** |
| --- | --- | --- |
| **Pubmed** | ((Unicompartmental Knee Arthroplasty) OR (Arthroplasty, Replacement, Partial Knee) OR (Arthroplasty, Unicompartmental Knee) OR (Knee Arthroplasty, Unicompartmental) OR (Unicompartmental Knee Replacement) OR (Knee Replacement, Unicompartmental) OR (Partial Knee Replacement) OR (Knee Replacement, Partial) OR (Unicondylar Knee Replacement) OR (Knee Replacement, Unicondylar) OR (Unicondylar Knee Arthroplasty) OR (Arthroplasty, Unicondylar Knee) OR (Knee Arthroplasty, Unicondylar) OR (Partial Knee Arthroplasty) OR (Arthroplasty, Partial Knee) OR (Knee Arthroplasty, Partial) ) AND (MAKO) | 132 |
| **Web of Science** | ((Unicompartmental Knee Arthroplasty) OR (Arthroplasty, Replacement, Partial Knee) OR (Arthroplasty, Unicompartmental Knee) OR (Knee Arthroplasty, Unicompartmental) OR (Unicompartmental Knee Replacement) OR (Knee Replacement, Unicompartmental) OR (Partial Knee Replacement) OR (Knee Replacement, Partial) OR (Unicondylar Knee Replacement) OR (Knee Replacement, Unicondylar) OR (Unicondylar Knee Arthroplasty) OR (Arthroplasty, Unicondylar Knee) OR (Knee Arthroplasty, Unicondylar) OR (Partial Knee Arthroplasty) OR (Arthroplasty, Partial Knee) OR (Knee Arthroplasty, Partial) ) AND (MAKO) | 64 |
| **Cochrane library** | ((Unicompartmental Knee Arthroplasty) OR (Arthroplasty, Replacement, Partial Knee) OR (Arthroplasty, Unicompartmental Knee) OR (Knee Arthroplasty, Unicompartmental) OR (Unicompartmental Knee Replacement) OR (Knee Replacement, Unicompartmental) OR (Partial Knee Replacement) OR (Knee Replacement, Partial) OR (Unicondylar Knee Replacement) OR (Knee Replacement, Unicondylar) OR (Unicondylar Knee Arthroplasty) OR (Arthroplasty, Unicondylar Knee) OR (Knee Arthroplasty, Unicondylar) OR (Partial Knee Arthroplasty) OR (Arthroplasty, Partial Knee) OR (Knee Arthroplasty, Partial) ) AND (MAKO) in All Text - (Word variations have been searched) | 10 |
| **Embase** | ('unicompartmental knee arthroplasty'/exp OR 'unicompartmental knee arthroplasty' OR (unicompartmental AND ('knee'/exp OR knee) AND ('arthroplasty'/exp OR arthroplasty)) OR 'arthroplasty, replacement, partial knee' OR (('arthroplasty,'/exp OR arthroplasty,) AND replacement, AND partial AND ('knee'/exp OR knee)) OR 'arthroplasty, unicompartmental knee' OR (('arthroplasty,'/exp OR arthroplasty,) AND unicompartmental AND ('knee'/exp OR knee)) OR 'knee arthroplasty, unicompartmental' OR (('knee'/exp OR knee) AND ('arthroplasty,'/exp OR arthroplasty,) AND unicompartmental) OR 'unicompartmental knee replacement'/exp OR 'unicompartmental knee replacement' OR (unicompartmental AND ('knee'/exp OR knee) AND ('replacement'/exp OR replacement)) OR 'knee replacement, unicompartmental' OR (('knee'/exp OR knee) AND replacement, AND unicompartmental) OR 'partial knee replacement'/exp OR 'partial knee replacement' OR (partial AND ('knee'/exp OR knee) AND ('replacement'/exp OR replacement)) OR 'knee replacement, partial' OR (('knee'/exp OR knee) AND replacement, AND partial) OR 'unicondylar knee replacement'/exp OR 'unicondylar knee replacement' OR (unicondylar AND ('knee'/exp OR knee) AND ('replacement'/exp OR replacement)) OR 'knee replacement, unicondylar' OR (('knee'/exp OR knee) AND replacement, AND unicondylar) OR 'unicondylar knee arthroplasty'/exp OR 'unicondylar knee arthroplasty' OR (unicondylar AND ('knee'/exp OR knee) AND ('arthroplasty'/exp OR arthroplasty)) OR 'arthroplasty, unicondylar knee' OR (('arthroplasty,'/exp OR arthroplasty,) AND unicondylar AND ('knee'/exp OR knee)) OR 'knee arthroplasty, unicondylar' OR (('knee'/exp OR knee) AND ('arthroplasty,'/exp OR arthroplasty,) AND unicondylar) OR 'partial knee arthroplasty'/exp OR 'partial knee arthroplasty' OR (partial AND ('knee'/exp OR knee) AND ('arthroplasty'/exp OR arthroplasty)) OR 'arthroplasty, partial knee' OR (('arthroplasty,'/exp OR arthroplasty,) AND partial AND ('knee'/exp OR knee)) OR 'knee arthroplasty, partial' OR (('knee'/exp OR knee) AND ('arthroplasty,'/exp OR arthroplasty,) AND partial)) AND ('mako'/exp OR mako) | 137 |
| **ClinicalTrials.gov** | ((Unicompartmental Knee Arthroplasty) OR (Arthroplasty, Replacement, Partial Knee) OR (Arthroplasty, Unicompartmental Knee) OR (Knee Arthroplasty, Unicompartmental) OR (Unicompartmental Knee Replacement) OR (Knee Replacement, Unicompartmental) OR (Partial Knee Replacement) OR (Knee Replacement, Partial) OR (Unicondylar Knee Replacement) OR (Knee Replacement, Unicondylar) OR (Unicondylar Knee Arthroplasty) OR (Arthroplasty, Unicondylar Knee) OR (Knee Arthroplasty, Unicondylar) OR (Partial Knee Arthroplasty) OR (Arthroplasty, Partial Knee) OR (Knee Arthroplasty, Partial) ) AND (MAKO) | 9 |
| **Scopus** | ( TITLE-ABS-KEY ( total AND knee AND arthroplasty ) OR TITLE-ABS-KEY ( total AND knee AND replacement ) AND ALL ( navio ) ) | 152 |
| **China National Knowledge Infrastructure** | （全文：单髁(精确)）AND（全文：置换(精确)）AND（全文：膝(精确)）AND（全文：MAKO(精确)） | 155 |
| **Wanfang, China Biology Medicine Disc** | 全部:("单髁") and 全部:("置换") and 全部:("膝") and 全部:("MAKO") | 10 |
| **China Science and Technology Journal (CSTD) databases** | 任意字段=单髁 AND任意字段=置换 AND任意字段=膝 AND任意字段=MAKO | 9 |
