# Supplementary material for: Comparison of MAKO robotic-assisted and manual unicompartmental knee arthroplasty: a meta-analysis of radiographic precision and short-term functional results
Source: J Robot Surg. 2026 Mar 2;20(1):307. doi: 10.1007/s11701-026-03259-y (PMC12953483; doi:10.1007/s11701-026-03259-y)

The agreement was found to be κ = 0.9040 for the title/abstract screening phase and κ = 0.8670 for the full-text review phase


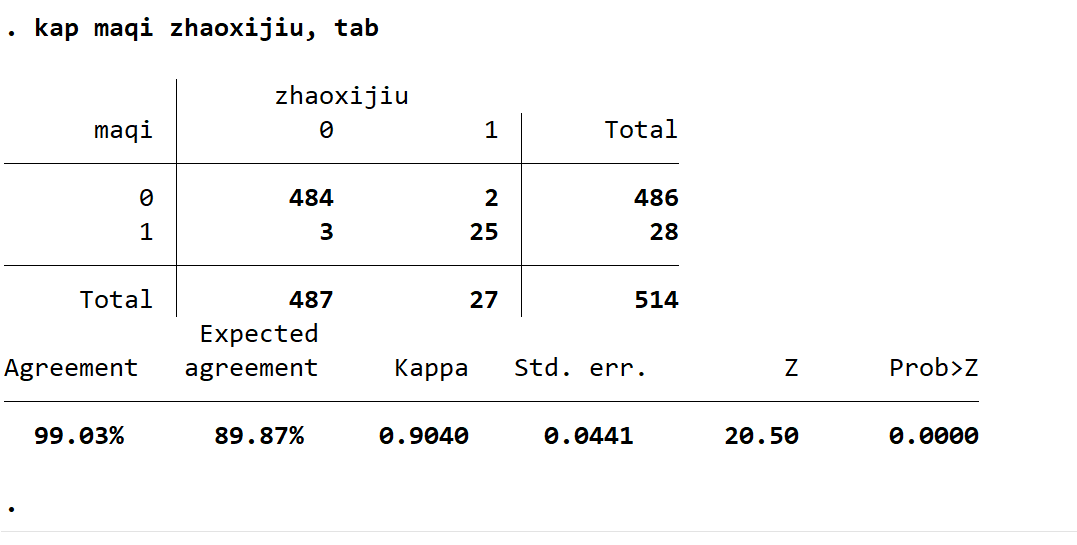


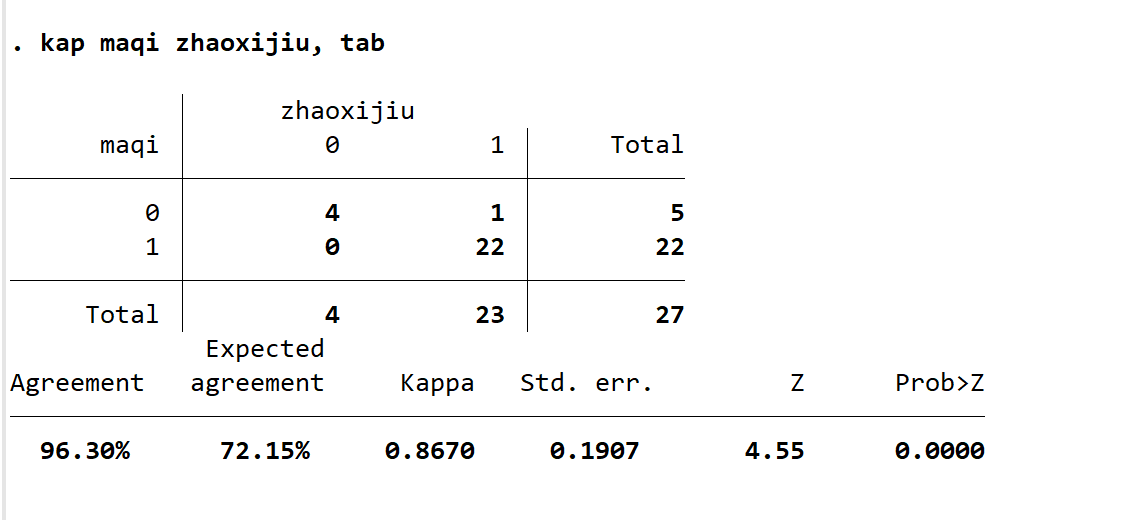

Supplement: Supplementary file 1 — Supplementary Material 1 [file 11701_2026_3259_MOESM1_ESM.zip › Supplementary Appendix/data2 kappa.docx]
