# Supplementary figures and images for: Comparison of MAKO robotic-assisted and manual unicompartmental knee arthroplasty: a meta-analysis of radiographic precision and short-term functional results
Source: J Robot Surg. 2026 Mar 2;20(1):307. doi: 10.1007/s11701-026-03259-y (PMC12953483; doi:10.1007/s11701-026-03259-y)

**TCCA egger test**


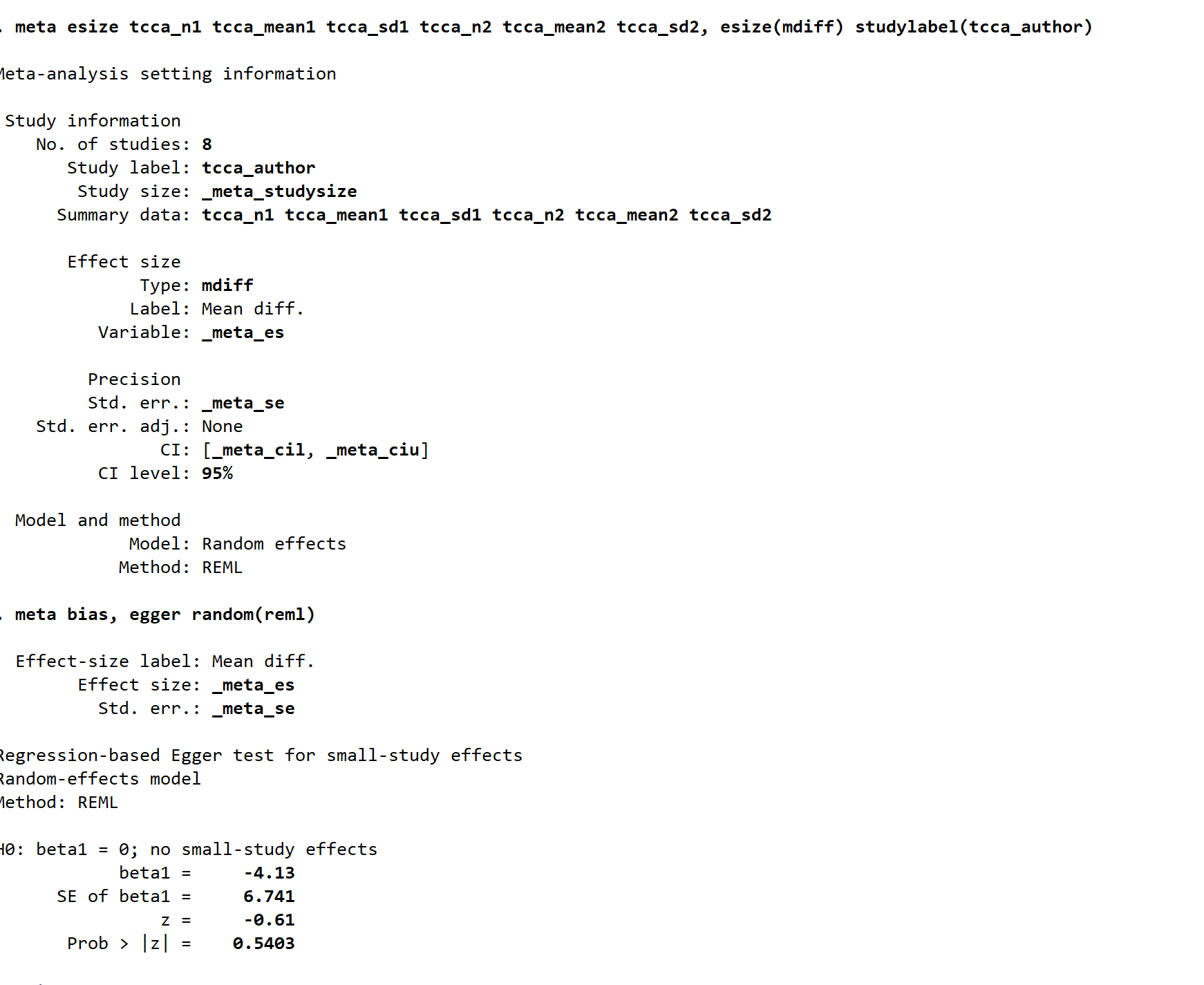


**Rr egger test**


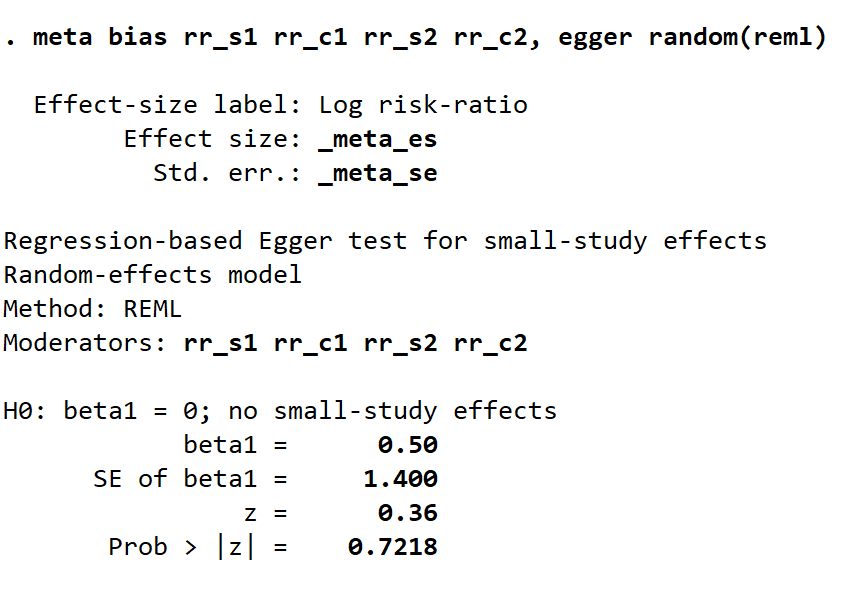


**CR Egger test**


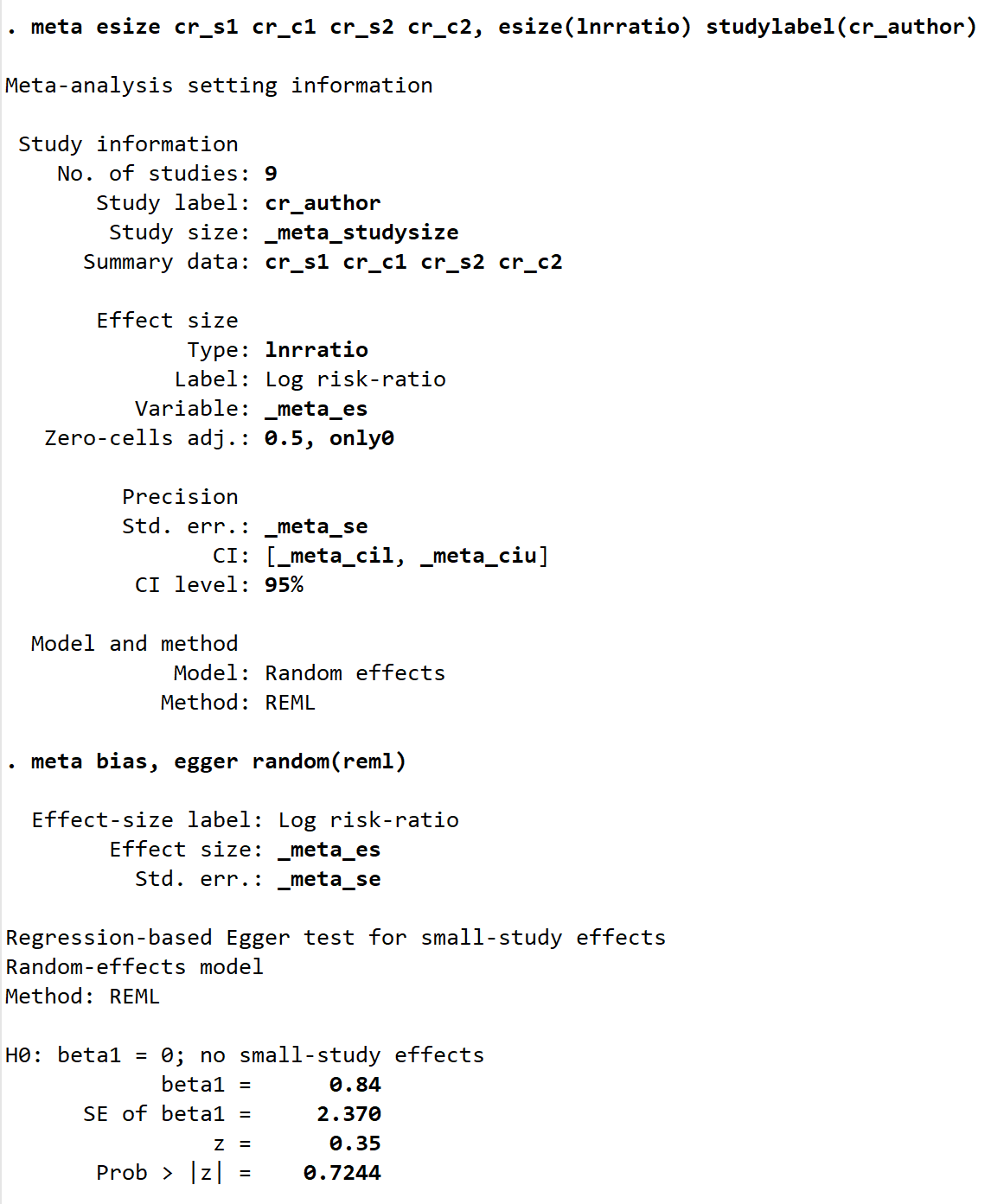

Supplement: Supplementary file 1 — Supplementary Material 1 [file 11701_2026_3259_MOESM1_ESM.zip › Supplementary Appendix/data 3 publication bias.docx]

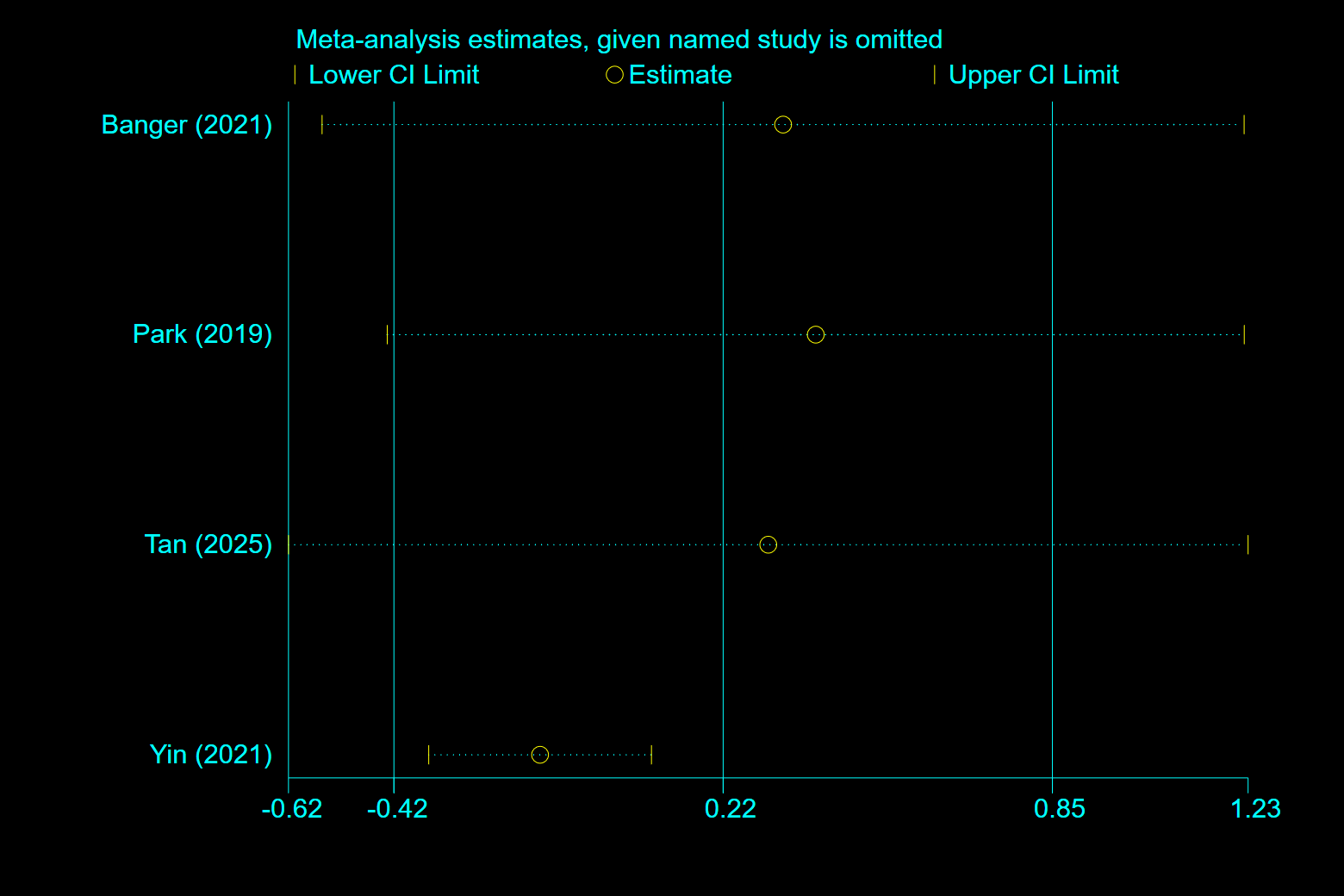

Supplement: Supplementary file 1 — Supplementary Material 1 [file 11701_2026_3259_MOESM1_ESM.zip › Supplementary Appendix/data 4 sensitivity analysis/akss.tif]

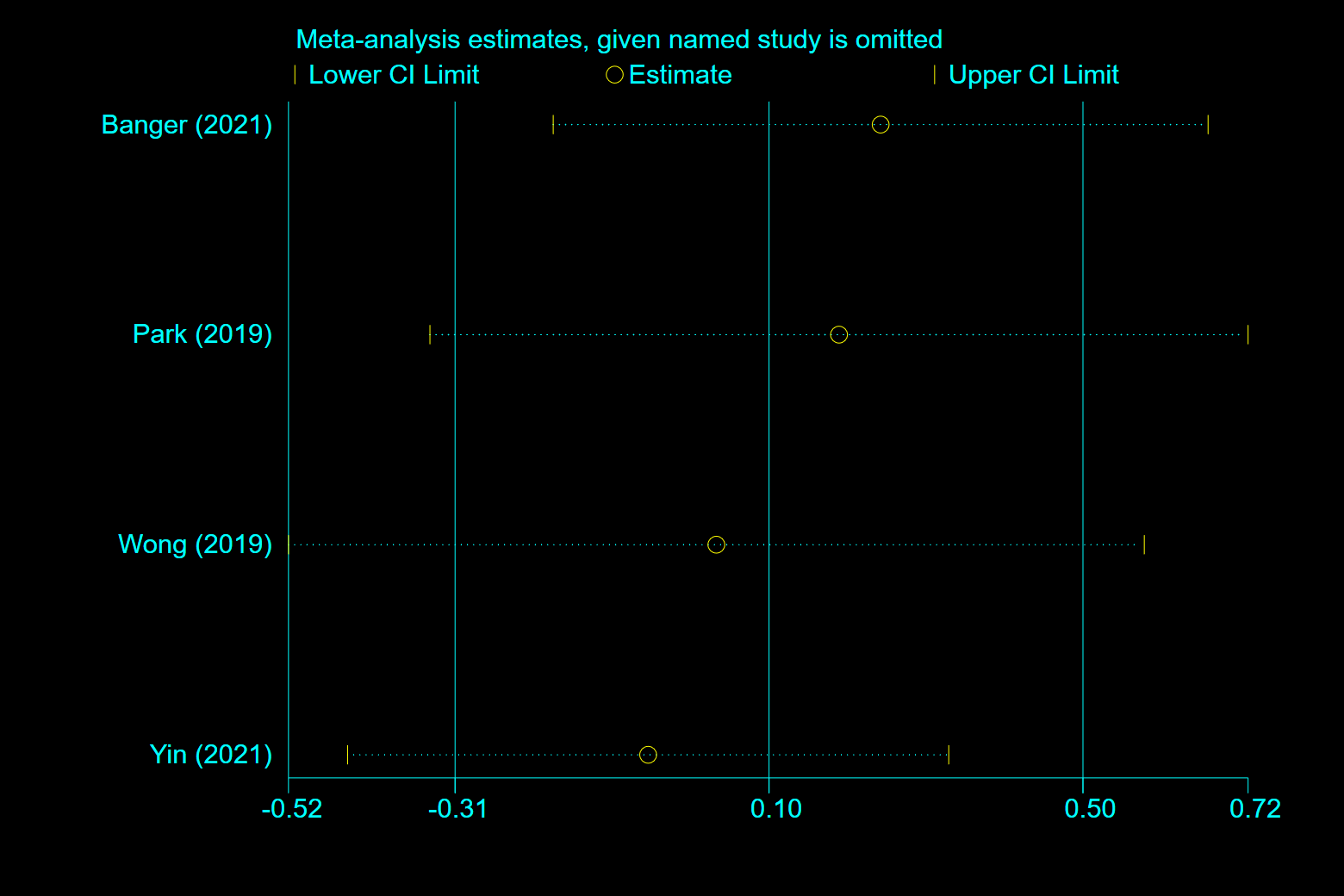

Supplement: Supplementary file 1 — Supplementary Material 1 [file 11701_2026_3259_MOESM1_ESM.zip › Supplementary Appendix/data 4 sensitivity analysis/akssf.tif]

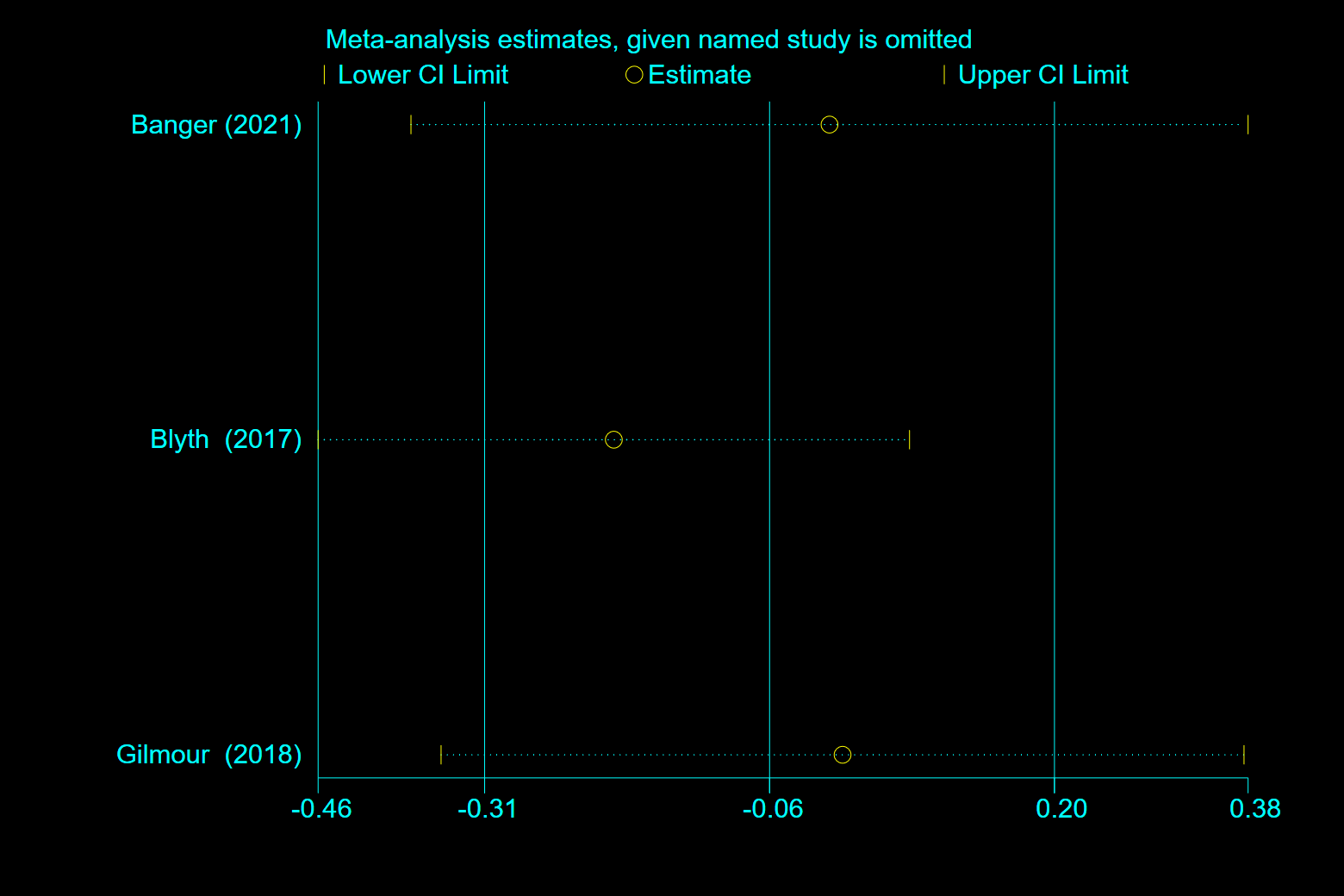

Supplement: Supplementary file 1 — Supplementary Material 1 [file 11701_2026_3259_MOESM1_ESM.zip › Supplementary Appendix/data 4 sensitivity analysis/aksst.tif]

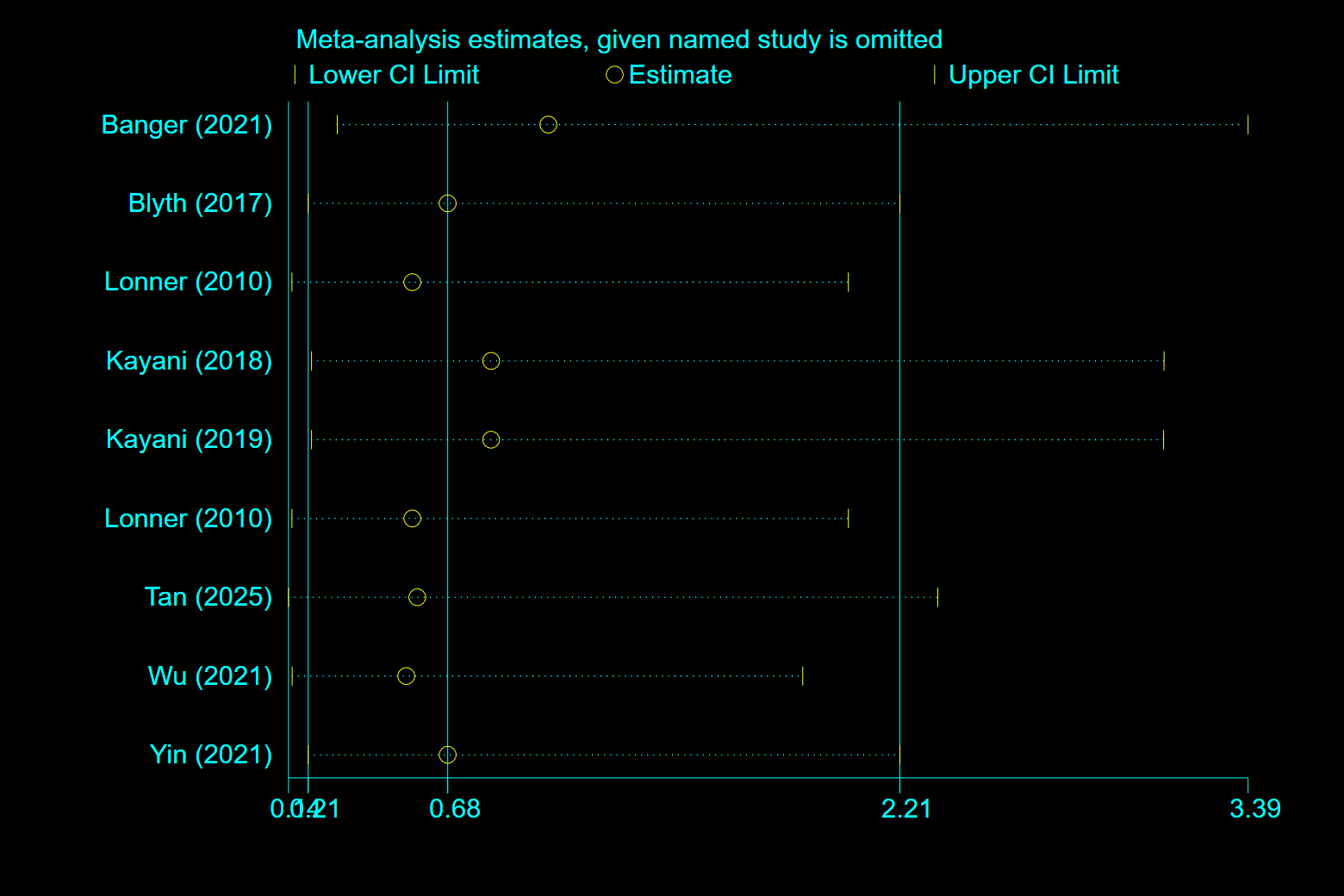

Supplement: Supplementary file 1 — Supplementary Material 1 [file 11701_2026_3259_MOESM1_ESM.zip › Supplementary Appendix/data 4 sensitivity analysis/cr.tif]

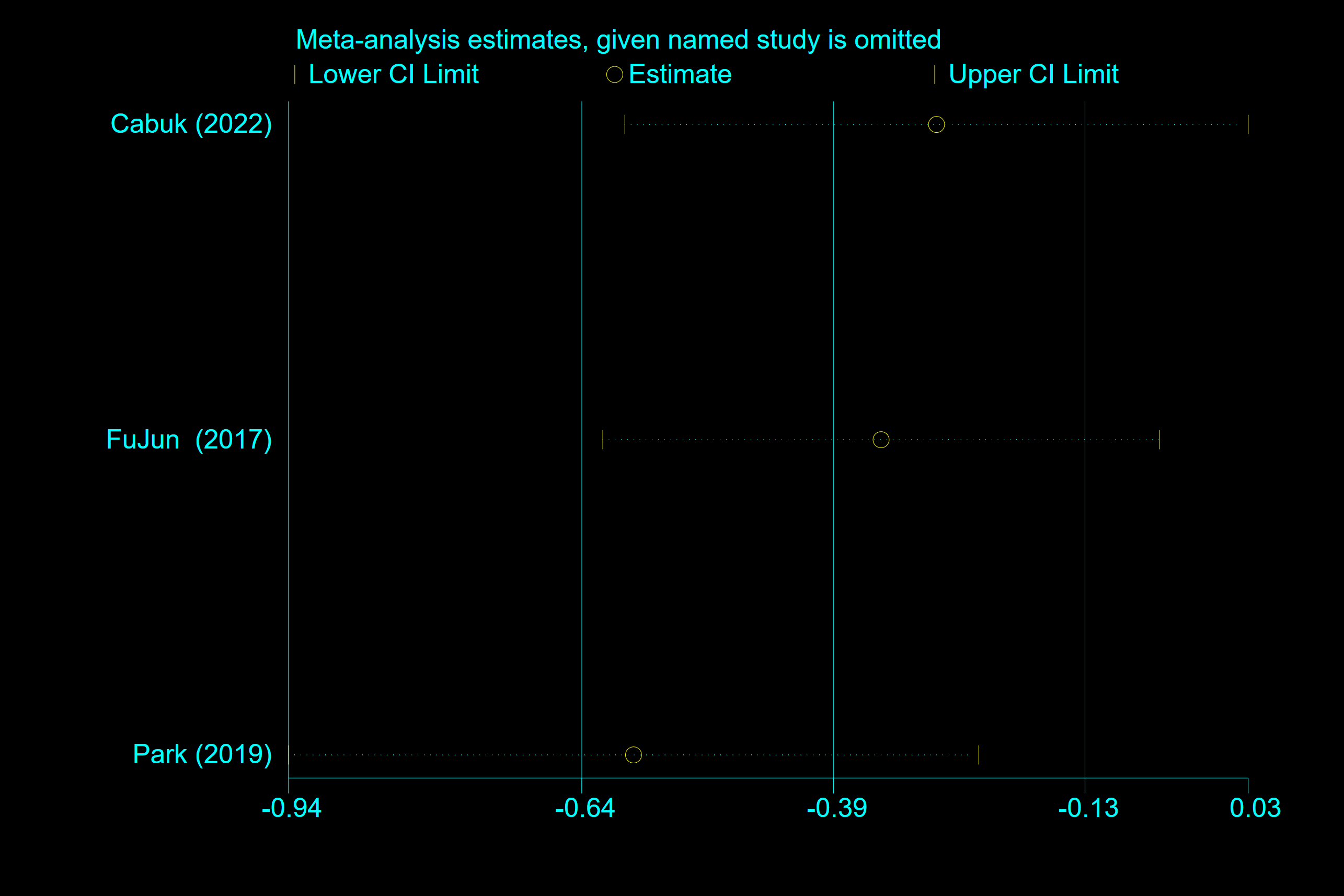

Supplement: Supplementary file 1 — Supplementary Material 1 [file 11701_2026_3259_MOESM1_ESM.zip › Supplementary Appendix/data 4 sensitivity analysis/fcca.tif]

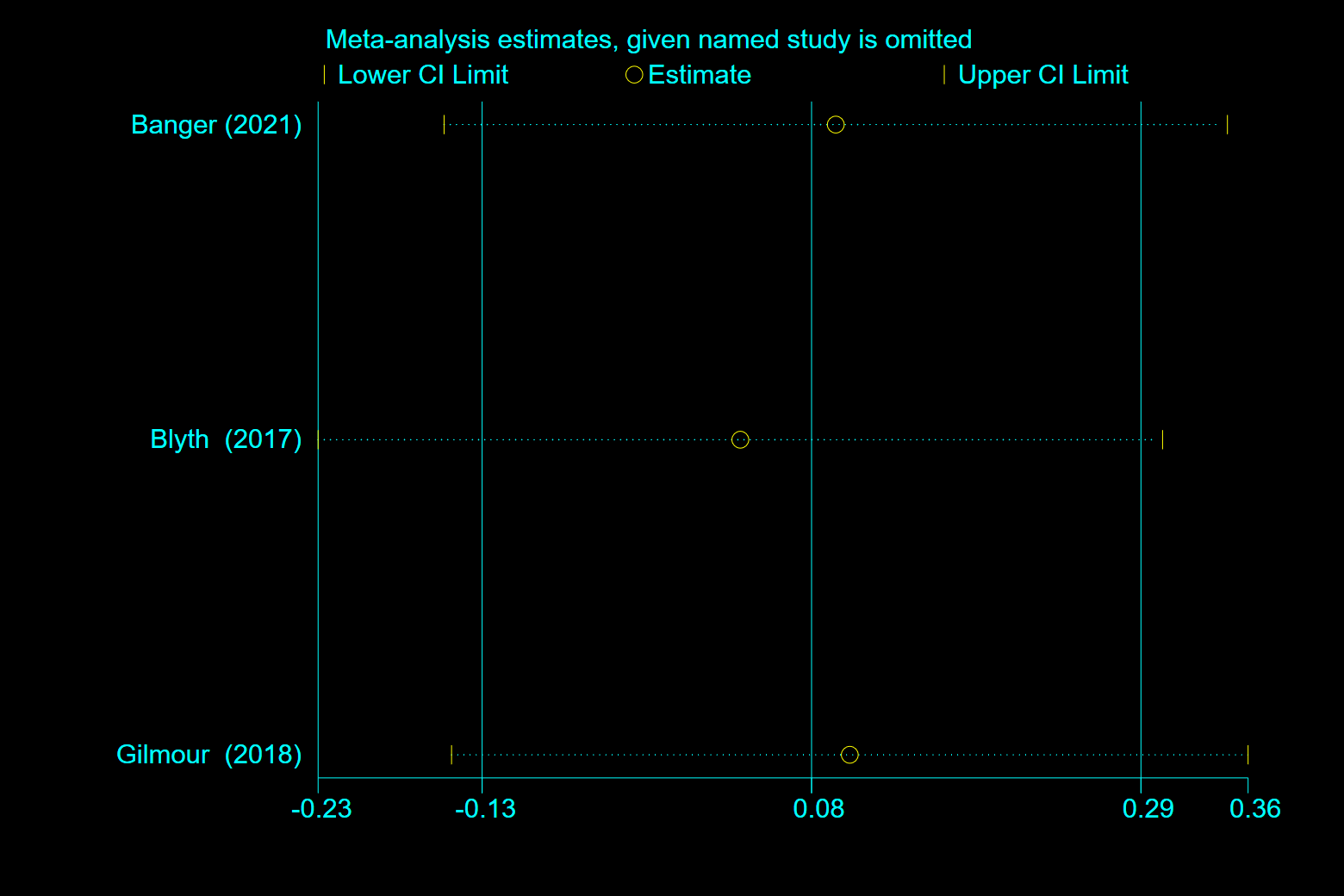

Supplement: Supplementary file 1 — Supplementary Material 1 [file 11701_2026_3259_MOESM1_ESM.zip › Supplementary Appendix/data 4 sensitivity analysis/fjs.tif]

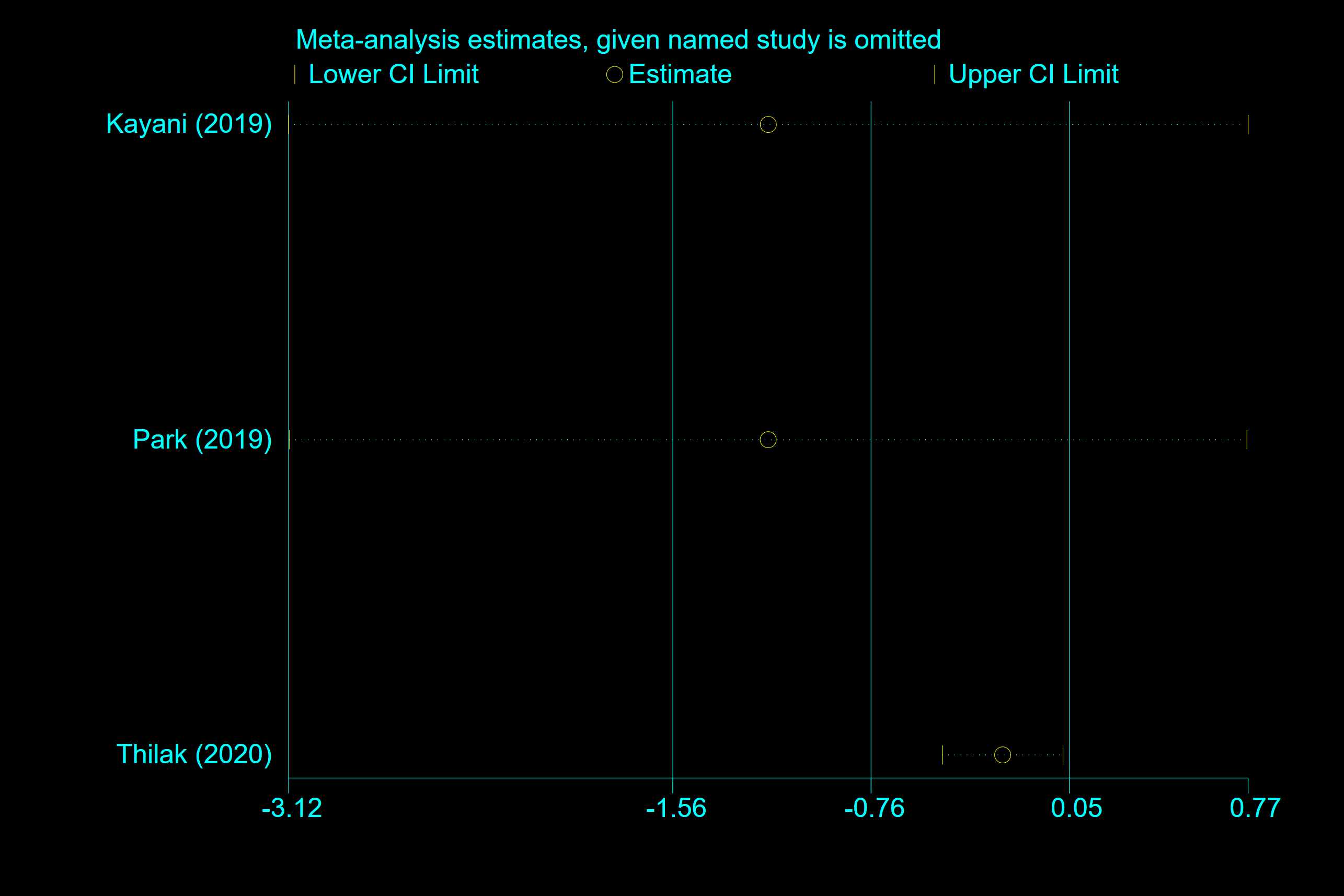

Supplement: Supplementary file 1 — Supplementary Material 1 [file 11701_2026_3259_MOESM1_ESM.zip › Supplementary Appendix/data 4 sensitivity analysis/mfta.tif]

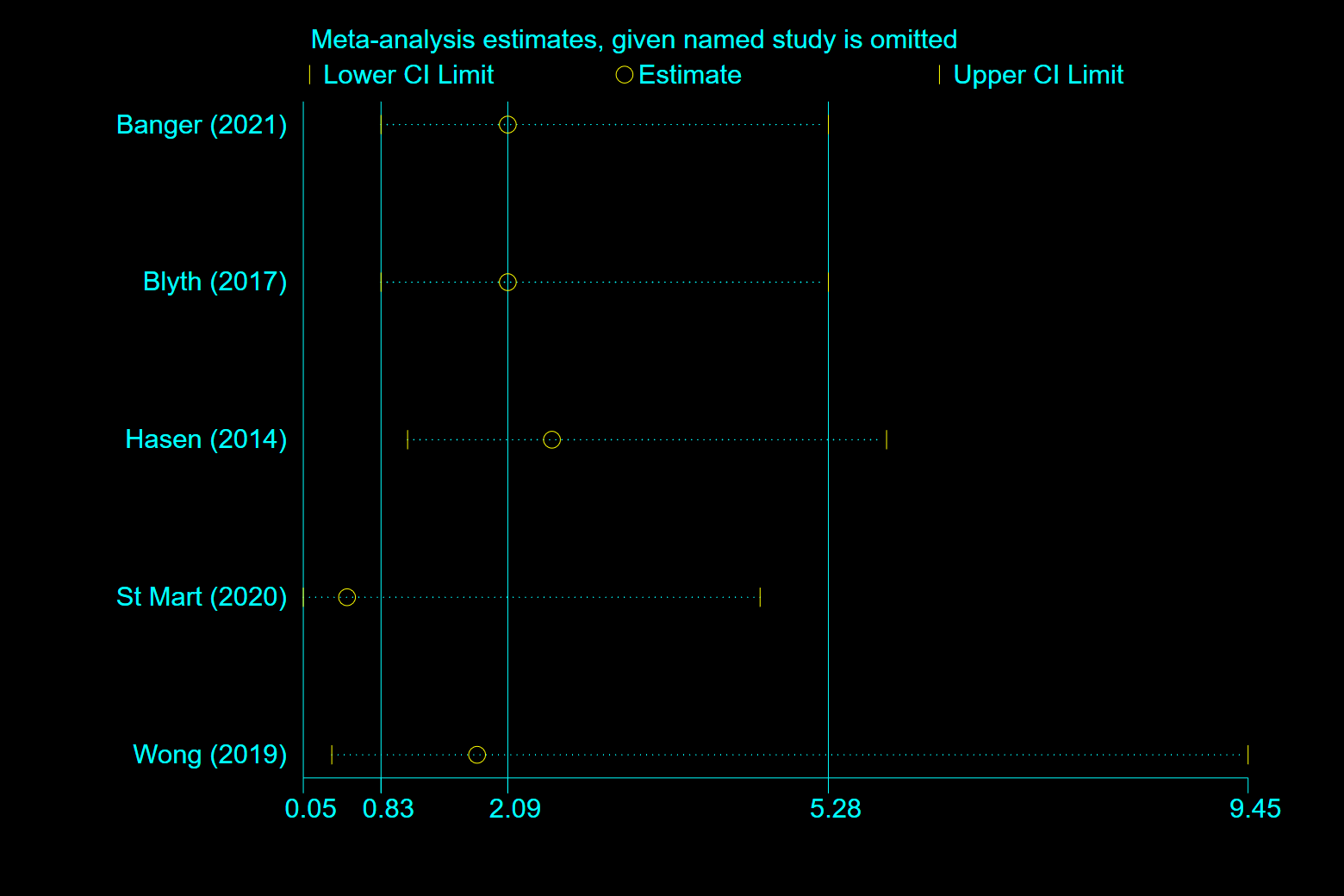

Supplement: Supplementary file 1 — Supplementary Material 1 [file 11701_2026_3259_MOESM1_ESM.zip › Supplementary Appendix/data 4 sensitivity analysis/not good/pjir.tif]

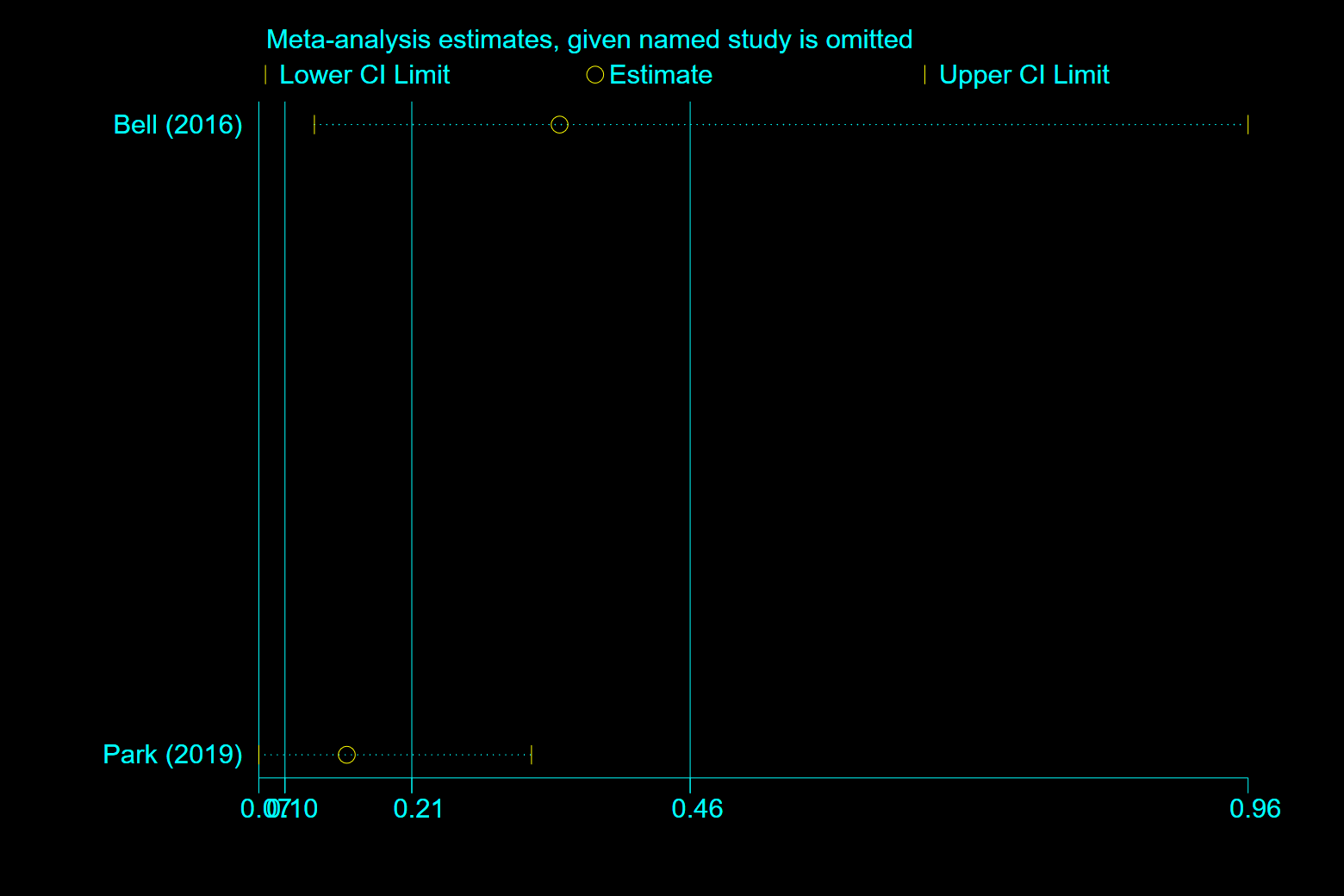

Supplement: Supplementary file 1 — Supplementary Material 1 [file 11701_2026_3259_MOESM1_ESM.zip › Supplementary Appendix/data 4 sensitivity analysis/ofcca.tif]

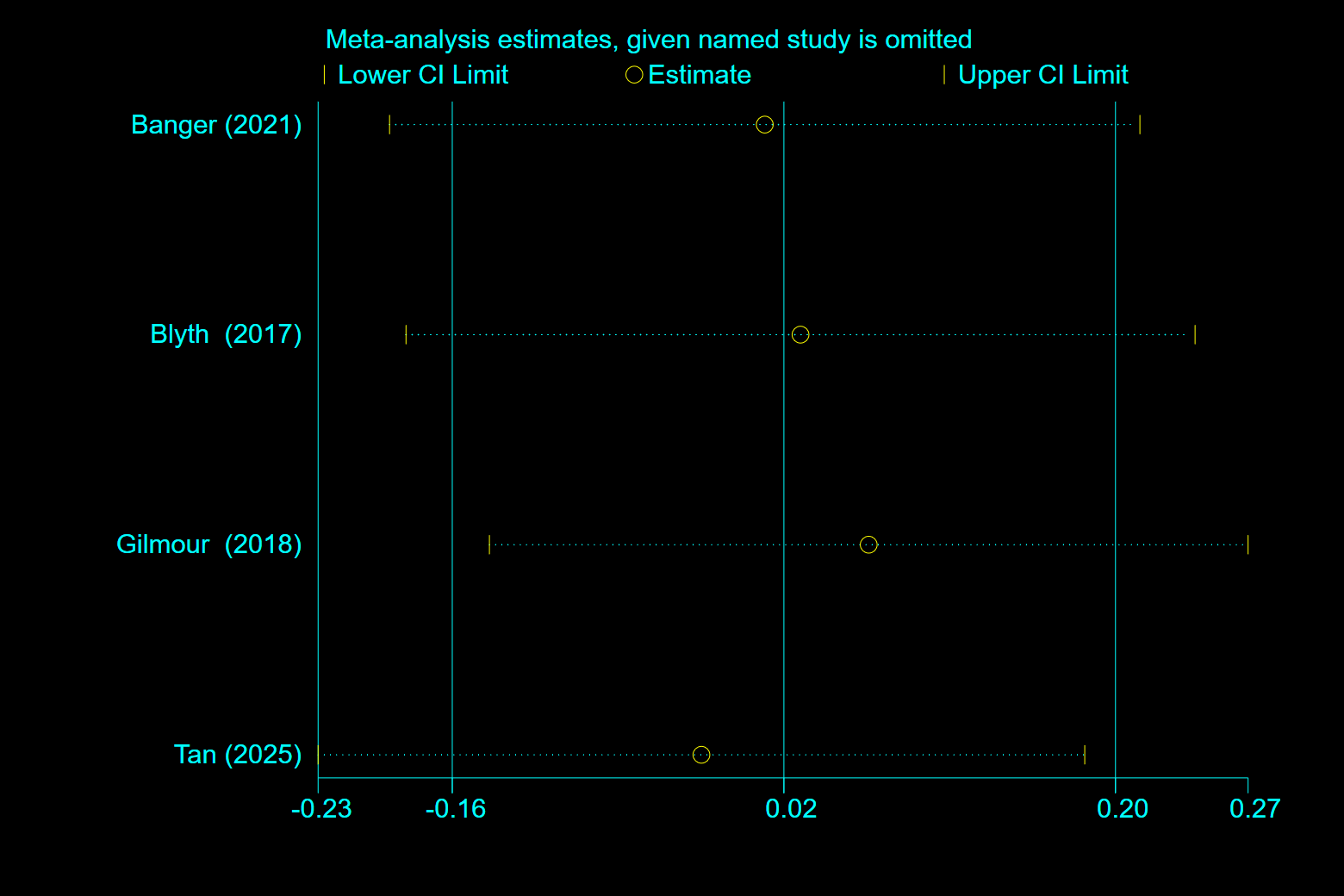

Supplement: Supplementary file 1 — Supplementary Material 1 [file 11701_2026_3259_MOESM1_ESM.zip › Supplementary Appendix/data 4 sensitivity analysis/oks.tif]

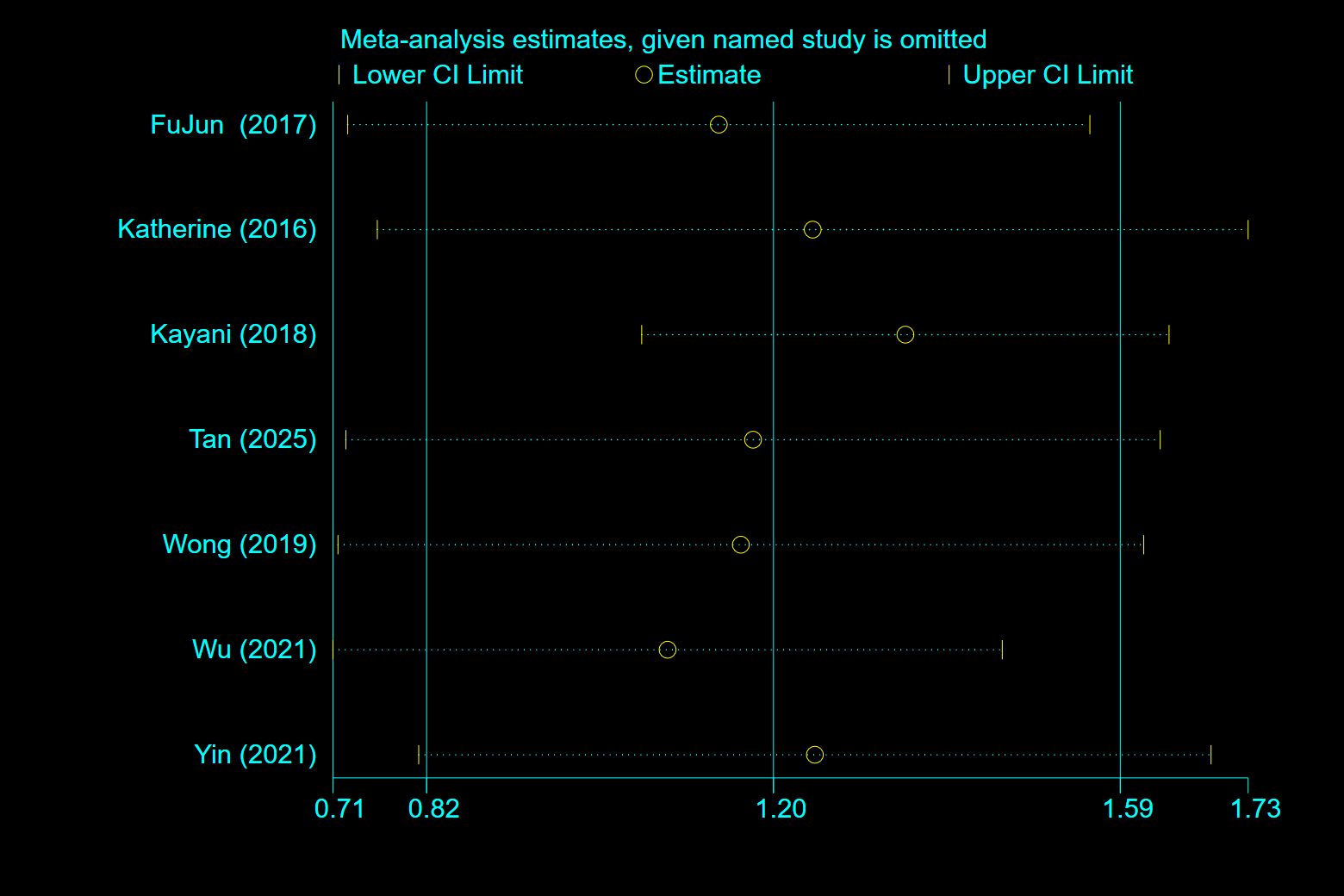

Supplement: Supplementary file 1 — Supplementary Material 1 [file 11701_2026_3259_MOESM1_ESM.zip › Supplementary Appendix/data 4 sensitivity analysis/op.tif]

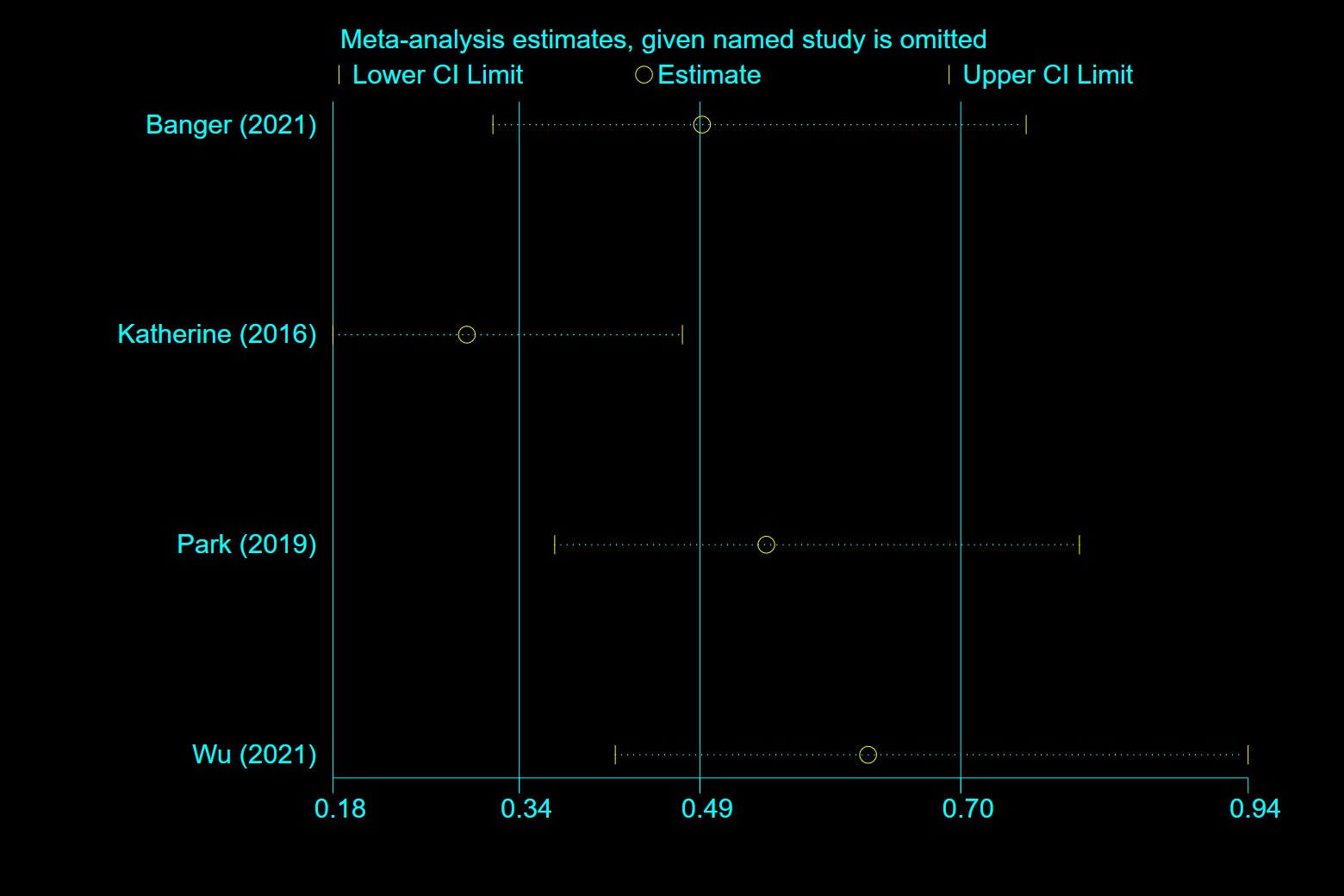

Supplement: Supplementary file 1 — Supplementary Material 1 [file 11701_2026_3259_MOESM1_ESM.zip › Supplementary Appendix/data 4 sensitivity analysis/otcca.tif]

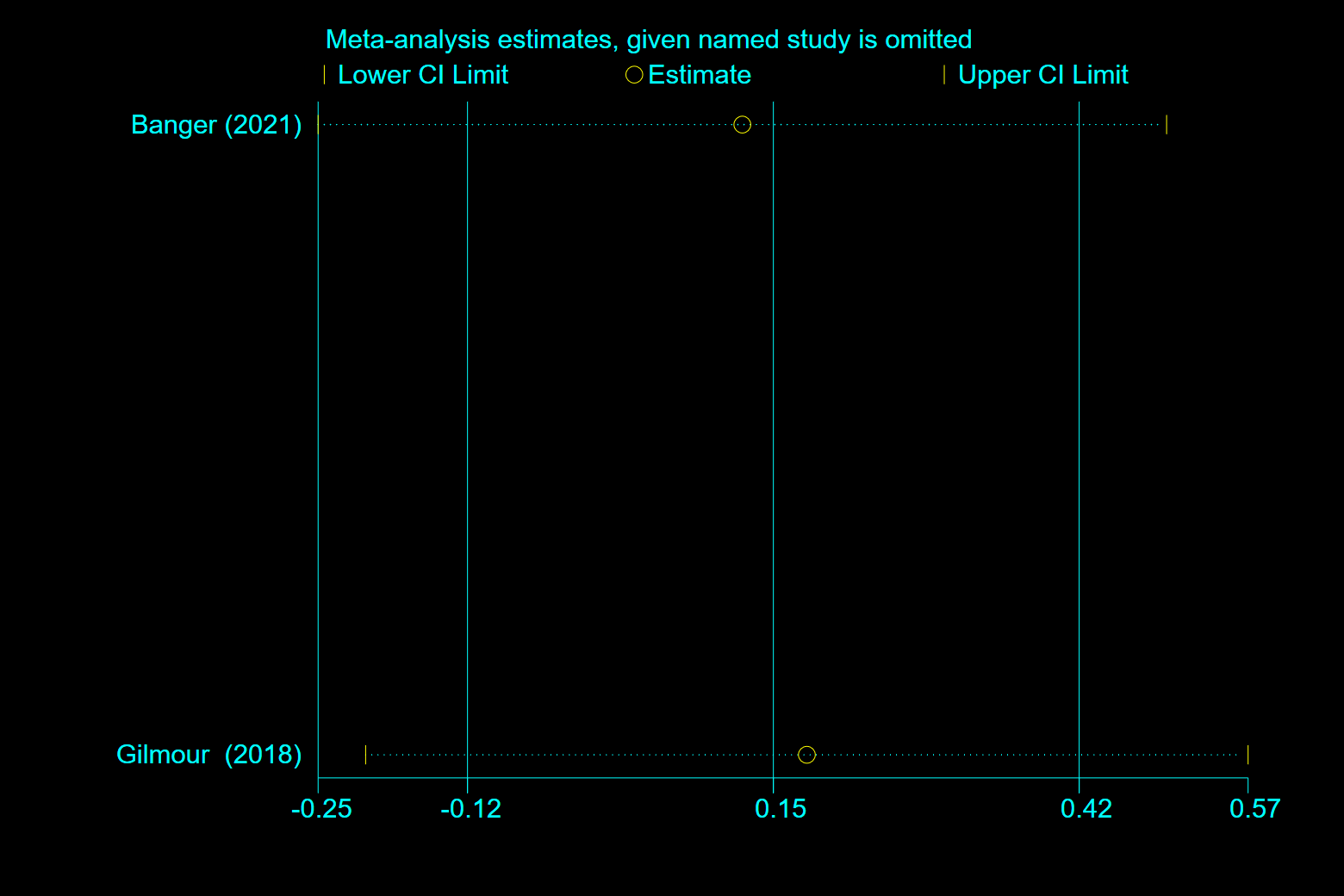

Supplement: Supplementary file 1 — Supplementary Material 1 [file 11701_2026_3259_MOESM1_ESM.zip › Supplementary Appendix/data 4 sensitivity analysis/pcs.tif]

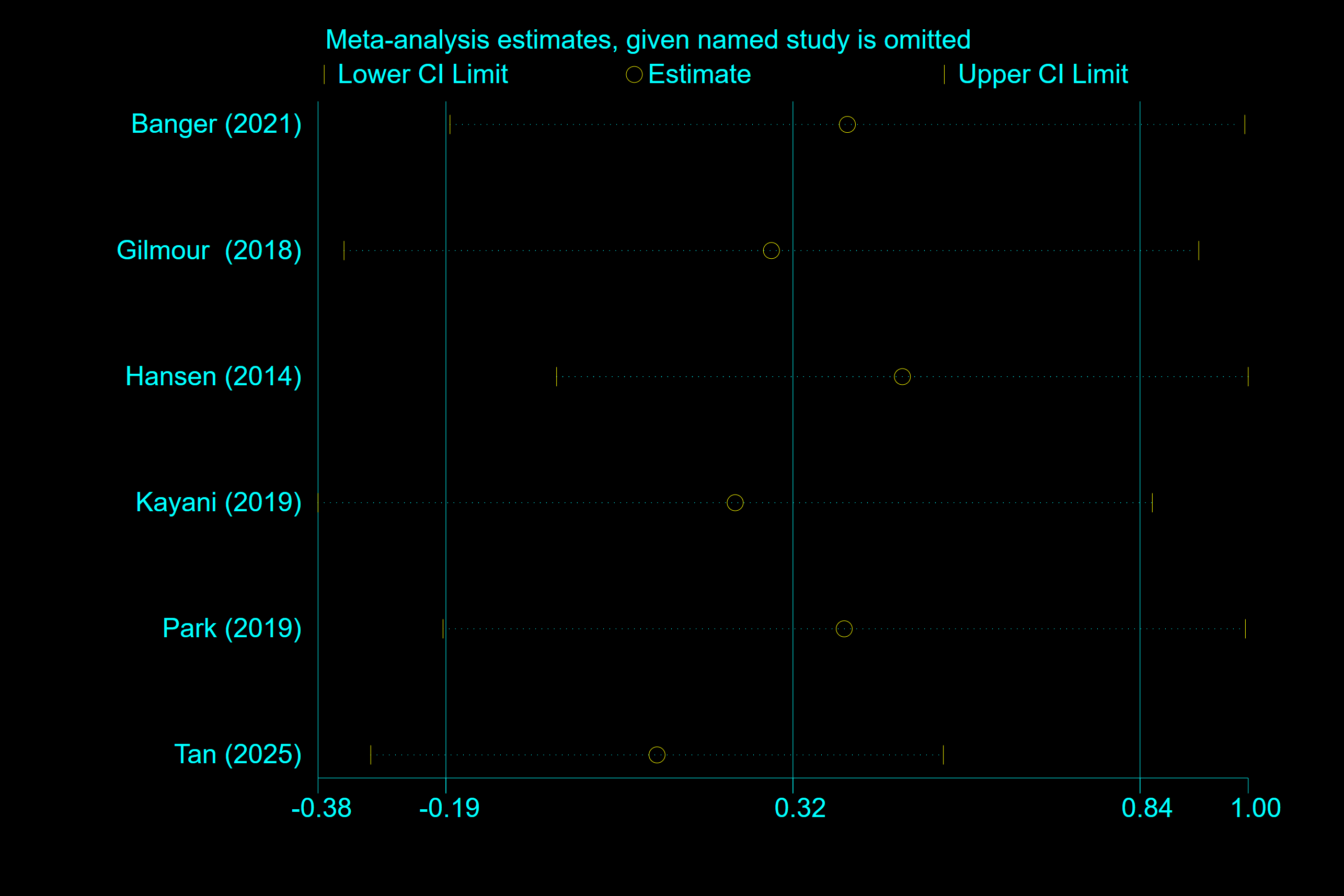

Supplement: Supplementary file 1 — Supplementary Material 1 [file 11701_2026_3259_MOESM1_ESM.zip › Supplementary Appendix/data 4 sensitivity analysis/rom.tif]

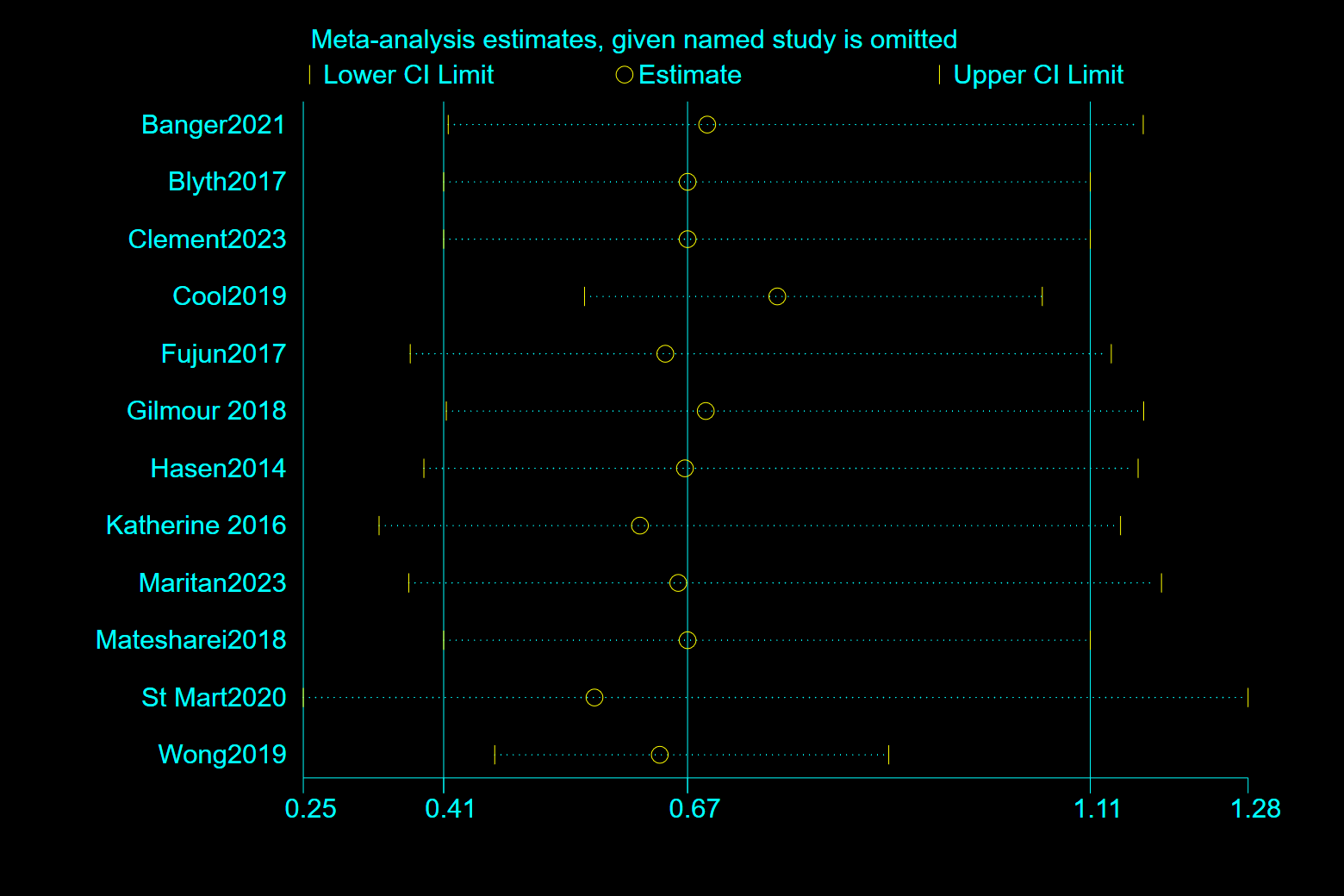

Supplement: Supplementary file 1 — Supplementary Material 1 [file 11701_2026_3259_MOESM1_ESM.zip › Supplementary Appendix/data 4 sensitivity analysis/rr.tif]

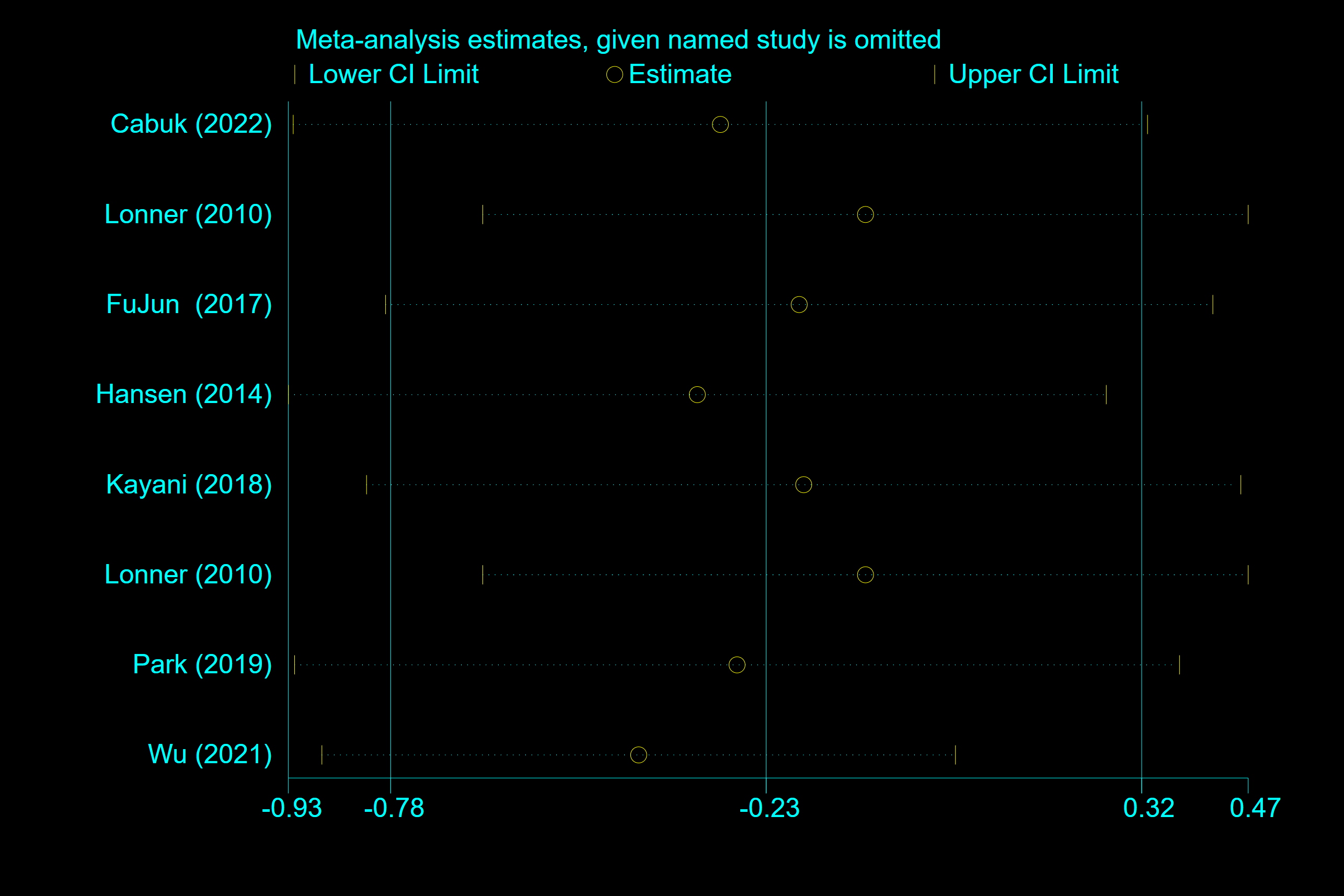

Supplement: Supplementary file 1 — Supplementary Material 1 [file 11701_2026_3259_MOESM1_ESM.zip › Supplementary Appendix/data 4 sensitivity analysis/tcca.tif]

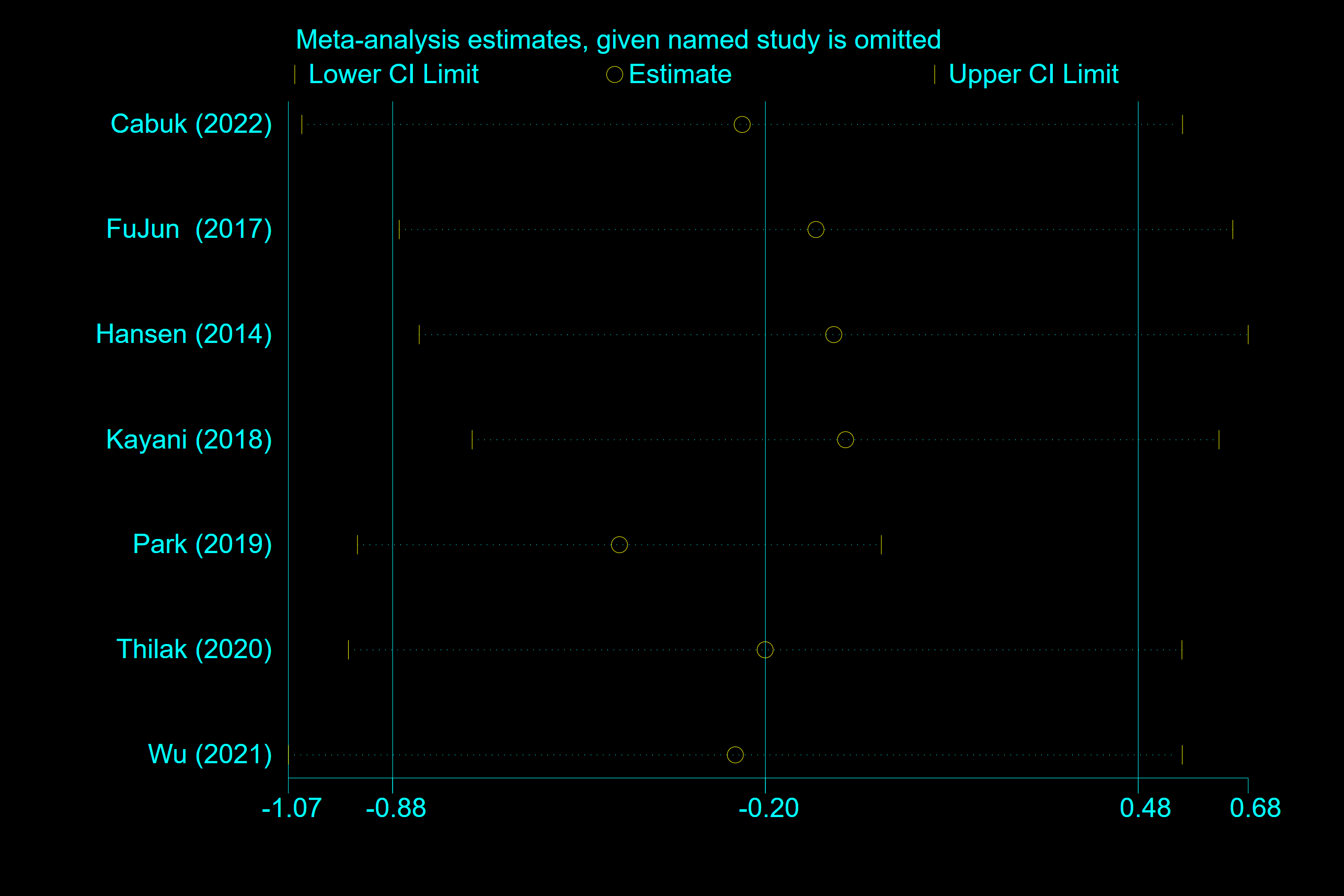

Supplement: Supplementary file 1 — Supplementary Material 1 [file 11701_2026_3259_MOESM1_ESM.zip › Supplementary Appendix/data 4 sensitivity analysis/tcpt.tif]

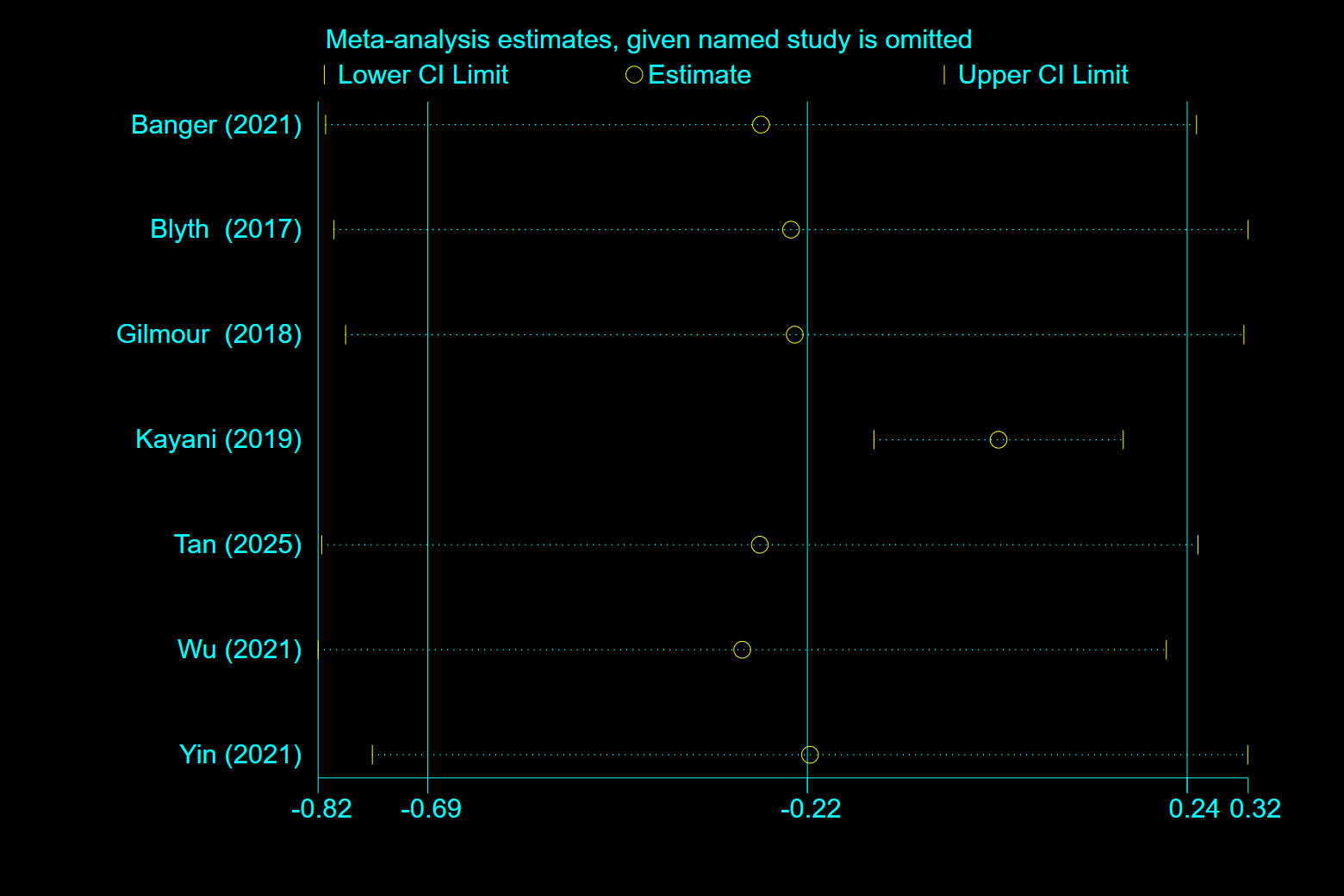

Supplement: Supplementary file 1 — Supplementary Material 1 [file 11701_2026_3259_MOESM1_ESM.zip › Supplementary Appendix/data 4 sensitivity analysis/vas.tif]
